# Mitochondrial ferritin alleviates ferroptosis in a kainic acid‐induced mouse epilepsy model by regulating iron homeostasis: Involvement of nuclear factor erythroid 2‐related factor 2

**DOI:** 10.1111/cns.14663

**Published:** 2024-03-04

**Authors:** Yu Song, Mengjiao Gao, Boyang Wei, Xiaowei Huang, Zeyu Yang, Junjie Zou, Yanwu Guo

**Affiliations:** ^1^ Department of Functional Neurosurgery, Neurosurgery Center, The National Key Clinical Specialty, Guangdong Provincial Key Laboratory on Brain Function Repair and Regeneration, The Neurosurgery Institute of Guangdong Province, Zhujiang Hospital Southern Medical University Guangzhou Guangdong China; ^2^ Department of Cerebrovascular Surgery, Neurosurgery Center, The National Key Clinical Specialty, The Engineering Technology Research Center of Education Ministry of China on Diagnosis and Treatment of Cerebrovascular Disease, Guangdong Provincial Key Laboratory on Brain Function Repair and Regeneration, The Neurosurgery Institute of Guangdong Province, Zhujiang Hospital Southern Medical University Guangzhou China; ^3^ Dongguan University of Technology Dongguan China; ^4^ School of Materials Science and Engineering, Key Laboratory for Polymeric Composite and Functional Materials of Ministry of Education Sun Yat‐sen University Guangzhou China

**Keywords:** epilepsy, Ferroptosis, FtMt, iron homeostasis, Nrf2

## Abstract

**Background:**

Epilepsy is a widespread and chronic disease of the central nervous system caused by a variety of factors. Mitochondrial ferritin (FtMt) refers to ferritin located within the mitochondria that may protect neurons against oxidative stress by binding excess free iron ions in the cytoplasm. However, the potential role of FtMt in epilepsy remains unclear. We aimed to investigate whether FtMt and its related mechanisms can regulate epilepsy by modulating ferroptosis.

**Methods:**

Three weeks after injection of adeno‐associated virus (AAV) in the skull of adult male C57BL/6 mice, kainic acid (KA) was injected into the hippocampus to induce seizures. Primary hippocampal neurons were transfected with siRNA using a glutamate‐mediated epilepsy model. After specific treatments, Western blot analysis, immunofluorescence, EEG recording, transmission electron microscopy, iron staining, silver staining, and Nissl staining were performed.

**Results:**

At different time points after KA injection, the expression of FtMt protein in the hippocampus of mice showed varying degrees of increase. Knockdown of the *FtMt* gene by AAV resulted in an increase in intracellular free iron levels and a decrease in the function of iron transport‐related proteins, promoting neuronal ferroptosis and exacerbating epileptic brain activity in the hippocampus of seizure mice. Additionally, increasing the expression level of FtMt protein was achieved by AAV‐mediated upregulation of nuclear factor erythroid 2‐related factor 2 (*Nrf2*) gene in the hippocampus of seizure mice.

**Conclusions:**

In epilepsy, Nrf2 modulates ferroptosis by involving the expression of FtMt and may be a potential therapeutic mechanism of neuronal injury after epilepsy. Targeting this relevant process for treatment may be a therapeutic strategy to prevent epilepsy.

## Graphical abstract

Mitochondrial ferritin (FtMt) can alleviate ferroptosis in the hippocampus of epileptic mice, thereby reducing epileptic brain activity. This process is related to the involvement of FtMt in the regulation of iron homeostasis. Additionally, the relevant function of FtMt in epilepsy may be regulated by nuclear factor erythroid 2‐related factor 2 (Nrf2).

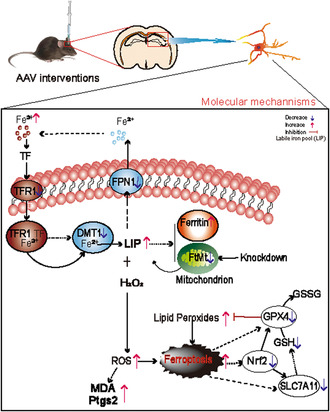

## 1 INTRODUCTION

Epilepsy is a common neurological disorder characterized by abnormal neuronal discharges resulting from neuronal damage, affecting over 70 million people worldwide.^1^, ^2^ Currently, research on the etiology of epilepsy mainly focuses on the cellular and molecular changes in the nervous system, such as inflammatory reactions, oxidative stress, and alterations in neurotransmitter levels. These mechanisms can lead to abnormalities in structures and functions such as the cerebral cortex and hippocampus.^3^ Due to the complex etiology and unclear mechanisms of epilepsy, the currently available treatment methods have not achieved satisfactory results. Therefore, we have explored the pathogenesis of epilepsy to provide new insights for treating epilepsy.

Ferroptosis is a form of cell death distinct from apoptosis, necrosis, and autophagy. It is characterized by the accumulation of lipid reactive oxygen species (ROS) due to excessive accumulation of free iron ions, which in turn leads to cellular damage.^4^, ^5^ Ferroptosis is involved in several pathological and physiological processes that occur in the nervous system and are associated with multiple neurological disorders, including traumatic brain injury (TBI), stroke, Alzheimer's, Parkinson's, Huntington's, and brain cancer.^6^, ^7^, ^8^, ^9^, ^10^, ^11^ In addition, ferroptosis of neurons has been demonstrated in the hippocampus of epileptic mice.^12^ Studies have illustrated that ferroptosis relies on the alteration of iron ion homeostasis, which is influenced by FtMt.^13^, ^14^, ^15^, ^16^, ^17^ However, the specific mechanisms and how regulating ferroptosis might help in treating epilepsy are unclear.

FtMt is an iron‐sequestering protein found in the mitochondria.^18^, ^19^, ^20^ The function of FtMt is to prevent mitochondrial and cell damage from iron‐induced oxidative stress by chelating excess free iron ions.^21^ In contrast to heavy chain ferritins (FtH) located in the cytoplasm, FtMt and FtH have fully conserved sequences and overlapping crystal structures in their iron oxidation centers and also share functional similarities. Unlike FtH, FtMt has no iron‐responsive elements (IRE) in its 5′UTR and is expressed only in organs with high oxygen demand. Therefore, the expression of FtMt may not depend on cellular iron levels, but rather its protective role in specific organs.^19^, ^20^, ^22^, ^23^, ^24^ Studies have demonstrated in a model of cerebral ischemia that modulation of FtMt within neurons affects ferroptosis.^17^ Therefore, the iron ion metabolism pathway mediated by FtMt may be a potential pathway for regulating neuronal ferroptosis in epilepsy.

Nrf2 is a key modulator in the antioxidant response and can improve oxidative stress by modulating downstream pathways or genes.^25^ Nrf2 alleviates seizures by inhibiting ferroptosis‐induced oxidative stress, however, the specific regulatory mechanism by which that occurs is unclear.^21^ Previous studies elucidated that Nrf2 mediates anti‐ferroptosis neuroprotection in TBI by regulating FtH and the ferritin light chain (FtL).^26^ However, the relationship between Nrf2 and FtMt in regulating free iron ion metabolism and ferroptosis is unknown. Therefore, Nrf2 may be involved in maintaining iron ion homeostasis and alleviating ferroptosis, potentially with the involvement of FtMt.

Here, we studied the regulation of FtMt by Nrf2 and its involvement in neuronal ferroptosis in epilepsy. We evaluated the expression levels of FtMt in animal and cellular models of epilepsy, and we demonstrated that downregulation of FtMt exacerbated iron ion dysregulation, leading to neuronal ferroptosis and epileptic brain activity in the hippocampus. Additionally, we investigated the regulatory mechanism of Nrf2 on FtMt in a mouse model of temporal lobe epilepsy (TLE) induced by kainic acid (KA). These findings may contribute to the discovery of new therapeutic approaches for epilepsy.

## 2 RESULTS

### 2.1 Expression and distribution of FtMt in the brains of epileptic mice

**FIGURE 1:**
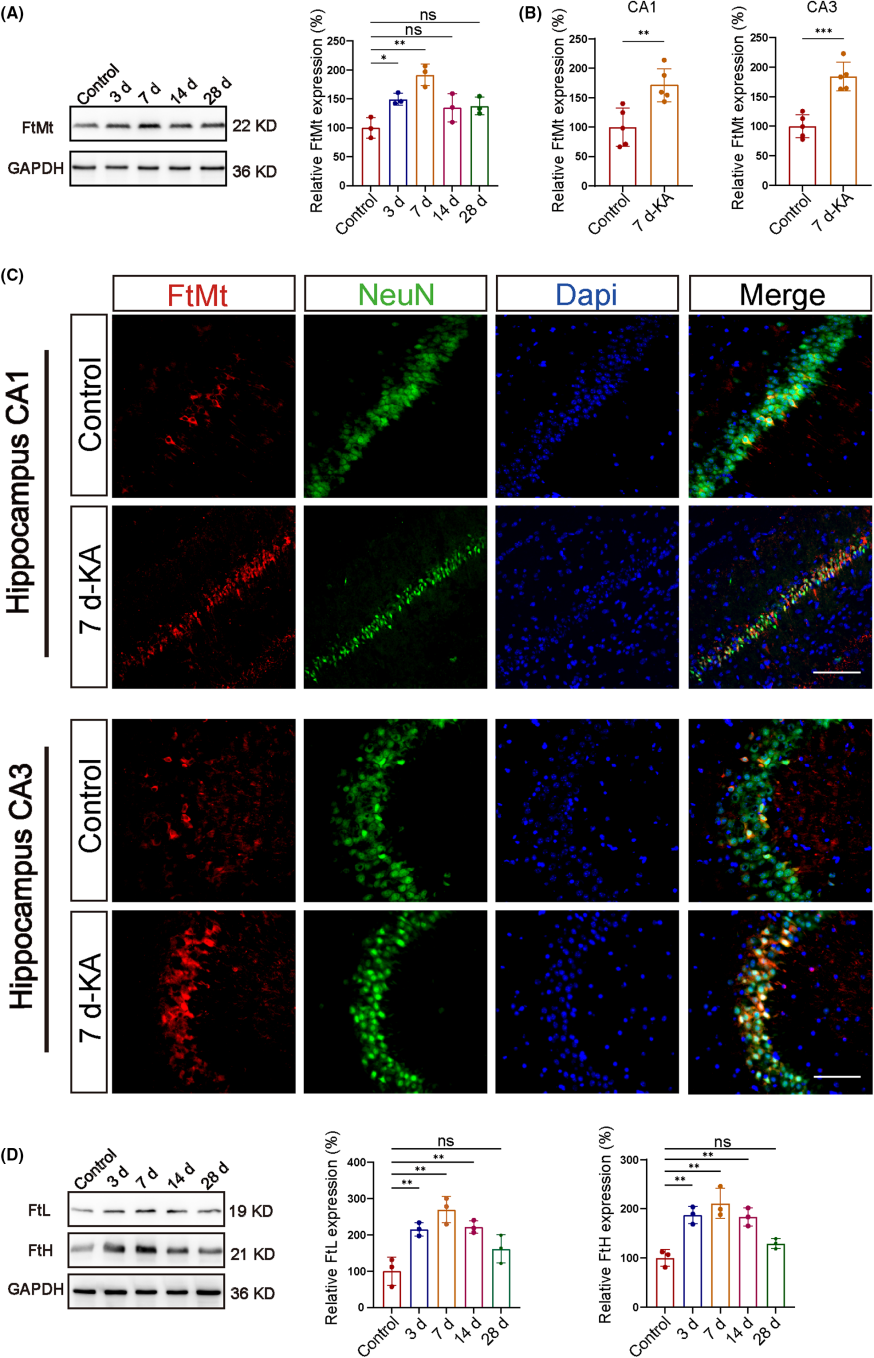
Expression and distribution of Mitochondrial ferritin (FtMt) during epilepsy. (A) The expression of FtMt was detected by western blotting, which showed that FtMt expression increased in epileptic mice on days 3, 7, 14, and 28 compared with the Control group (*n* = 3 in each group). (B, C) Changes in the expression of FtMt in neurons and its colocalization within neuronal cells in the hippocampal tissue of mice with epilepsy on the 7th day compared with the Control group (*n* = 5 in each group) (scale bar = 100 μm). (D) Protein expression of heavy chain ferritin (FtH) and light chain ferritin (FtL) was detected by western blotting, which showed that the levels increased in epileptic mice on days 3, 7, 14, and 28 compared with the Control group (*n* = 3 in each group). All data are depicted with SEM; *p < 0.05, **p < 0.01, ***p < 0.001 versus Control group, ns not significant.

After stereotactic injection of KA into the right hippocampi of mice, an electroencephalogram (EEG) was used to measure epileptic discharges. On days 3, 7, 14, and 28 after injection, brain tissue was collected. Videos were recorded on days 3, 7, 14, and 28 after injection (Figure S1F). Western blot analysis was used to compare FtMt protein expression in the right hippocampal tissue from KA‐induced epileptic mice to that of control mice. Hippocampal expression of FtMt was significantly up‐regulated in epileptic mice, with a peak on day 7 (Figure 1A). We also performed Western blot analysis to compare FtMt protein expression in the injected cortex of KA‐induced epilepsy mice with control mice. The expression of FtMt in the cortex was indistinguishable from that of the control group (Figure S1D). Immunofluorescence labeling was used to study changes in FtMt expression in the hippocampus of CA1, CA3, and DG on day 7 in epilepsy mice. The results showed that the FtMt in CA1 and CA3 regions was significantly increased in the epilepsy group, but there was no significant change in the DG area (Figure 1B,C; Figure S1E). To further determine the specific localization of FtMt during epilepsy, a double‐labeling immunofluorescence technique was used to detect the cellular localization of FtMt within epileptic brain tissue. In the hippocampus of KA‐induced TLE mice, FtMt colocalized within neuronal nuclei (NeuN; a neuronal marker), but not with glial fibrillary acidic protein (GFAP; an astrocytic marker) or ionized calcium‐binding adapter molecule 1 (Iba1; a microglial marker). The results indicated that FtMt was primarily expressed in neurons, with lower expression in astrocytes and microglia (Figure S2A). The FTH and FTL expression levels in the hippocampi of epileptic mice at different time points were also determined by western blotting. Hippocampal FtH and FtL expression were markedly increased in epileptic mice, with the highest levels observed on day 7 (Figure 1D).

### 2.2 Occurrence of ferroptosis in the mouse model of KA‐induced seizures

**FIGURE 2:**
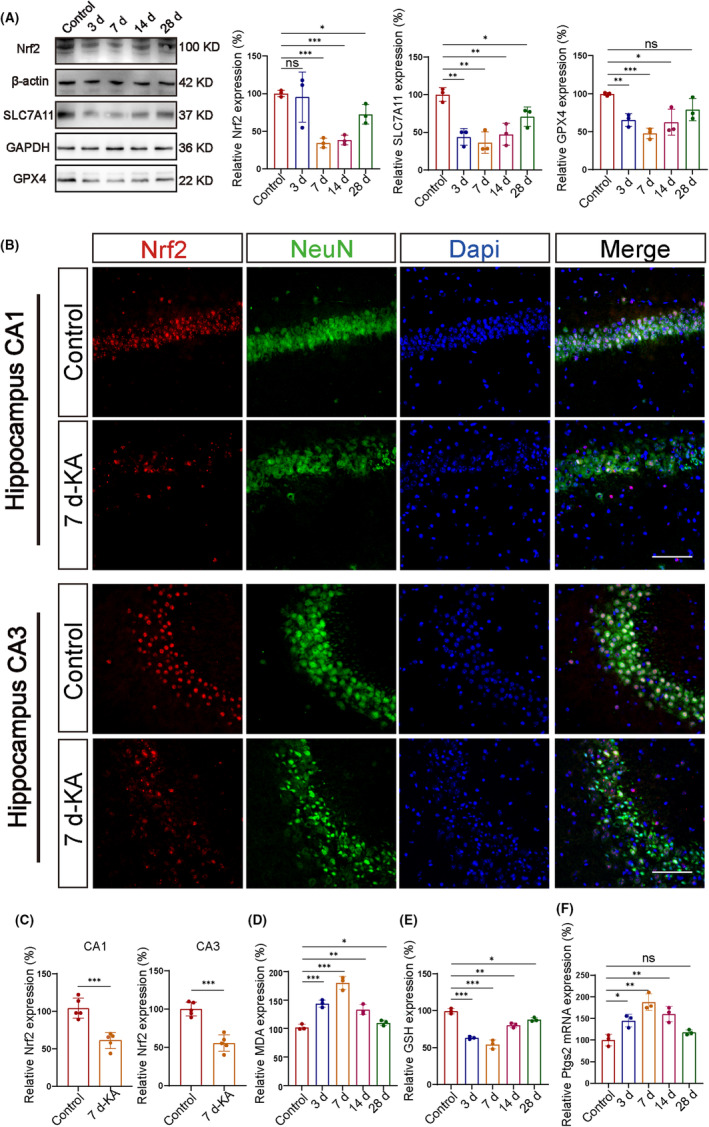
Ferroptosis occurs during KA‐induced seizures in mice. Western blot analysis of (A) glutathione peroxidase 4 (GPX4) and solute carrier family 7 member 11 (SLC7A11) decreased in epileptic mice compared with the Control group (*n* = 3 in each group). (B, C) The changes in Nrf2 levels in hippocampal neurons of epileptic mice on the 7th day compared with the Control group as well as its colocalization within neuronal cells (*n* = 5 in each group) (scale bar = 100 μm). (D–F) The expression levels of prostaglandin‐endoperoxide synthase 2 (*Ptgs2*) mRNA, glutathione (GSH), and malondialdehyde (MDA) in the hippocampus of epileptic mice at different time points (*n* = 3 in each group). All data are depicted with SEM; *p < 0.05, **p < 0.01, ***p < 0.001 versus Control group, ns not significant.

We collected the right hippocampal tissues of mice on days 3, 7, 14, and 28 after KA injection to study the effect of TLE on the hippocampus. Western blot detection of protein expression in the KA‐induced TLE model was compared with the control group. In the mouse model, a decrease in protein expression was observed through western blot analysis for solute carrier family 7 member 11 (SLC7A11) and glutathione peroxidase 4 (GPX4). Those proteins regulate ferroptosis by influencing lipid peroxidation^27^, ^28^ (Figure 2A). Immunofluorescence labeling was used to investigate changes in Nrf2 expression in the CA1 and CA3 hippocampal areas on day 7 mice with epilepsy. The results showed a significant decrease in the epileptic group (Figure 2B,C). To further determine the specific localization of Nrf2 during epilepsy, a double‐labefling immunofluorescence technique was used to detect the cellular localization of Nrf2 within epileptic brain tissue. In the hippocampus of KA‐induced TLE mice, Nrf2 colocalized within NeuN but not with GFAP or Iba1. The results indicated that Nrf2 was primarily expressed in neurons, with lower expression in astrocytes and microglia (Figure S2B). Similarly, we also measured the levels of lipid peroxidation‐related metabolites in cells. We used commercially available assay kits to measure the levels of malondialdehyde (MDA) and glutathione (GSH; Figure 2D,E). Studies have indicated that cyclooxygenase‐2, also called prostaglandin‐endoperoxide synthase 2 (*Ptgs2*), is recognized as a biomarker for ferroptosis.^17^ To quantify the *Ptgs2* mRNA expression in the hippocampus of epileptic mice, we performed a quantitative real‐time PCR assay (Figure 2F). The mRNA levels of Ptgs2 showed the most significant changes on day 7. Western blot analysis of iron‐related transport‐related proteins in epileptic mice showed decreased protein expression on day 7 (Figure S1A). Through transmission electron microscopy, we found that CA1 and CA3 mitochondrial atrophy and the double‐layer membrane structure were blurred and ruptured in the hippocampal tissue of mice on day 7 of epilepsy (Figure S1B). Additionally, through labeling with a dihydroethidium (DHE) probe, we observed the increased expression of ROS in the hippocampal tissue of epileptic mice compared with the control group (Figure S1C).

### 2.3 Occurrence of neuronal changes in the mouse model of KA‐induced seizures

**FIGURE 3:**
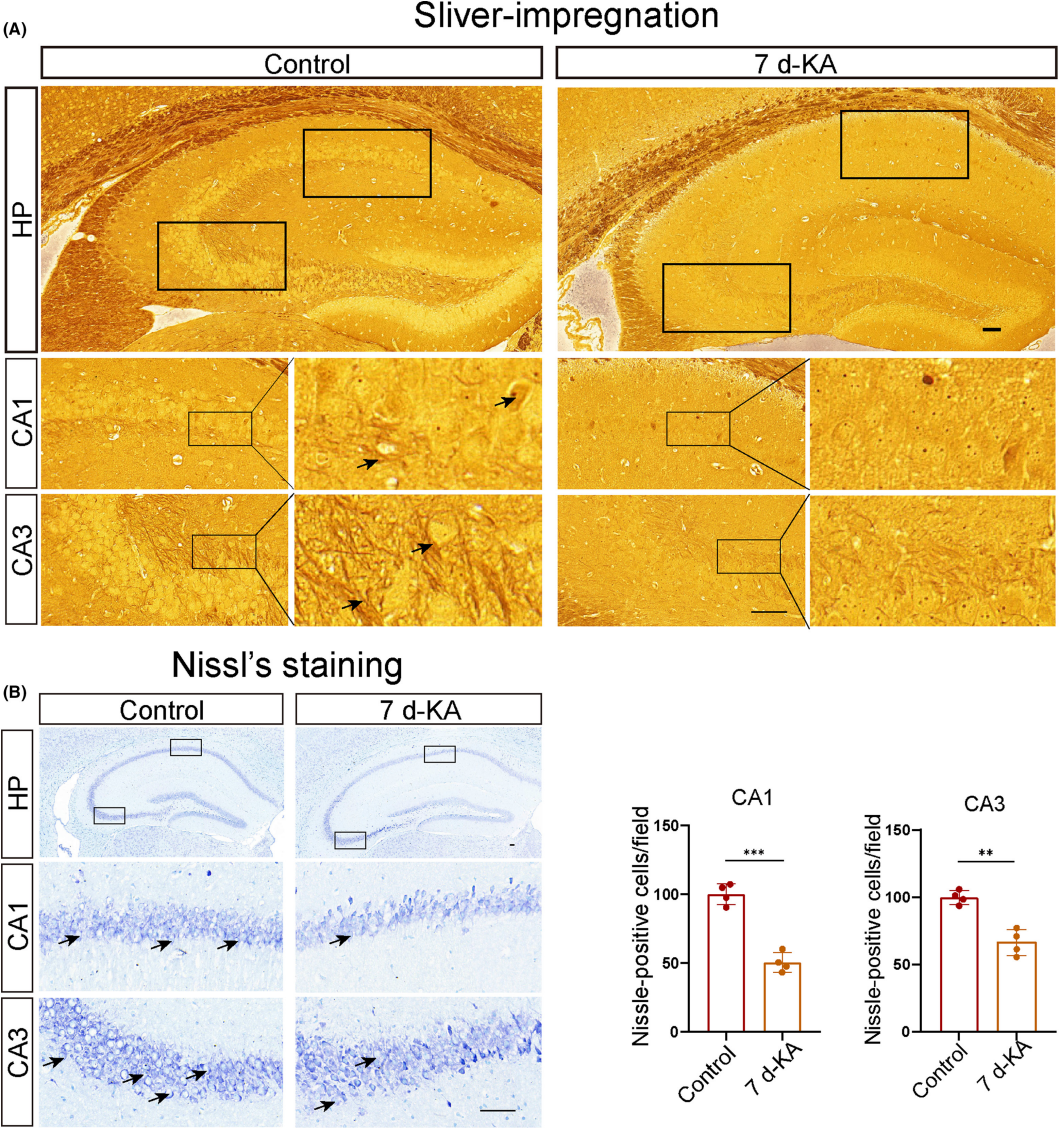
Occurrence of neuronal changes in the mouse model of KA‐induced seizures. (A) Silver staining of hippocampal tissue in the Control group and epileptic mice on the 7th day (scale bar = 100 μm). Arrows point to Nissl‐positive cells synapse. HP stands for hippocampus. (B) Nissl staining of hippocampal tissue in the Control group and epileptic mice on the 7th day (*n* = 4) (scale bar = 100 μm). Arrows point to Nissl‐positive cells. HP stands for hippocampus. All data are depicted with SEM; **p < 0.01, ***p < 0.001 versus Control group, ns not significant.

To investigate the effects of TLE on hippocampal neurons, we selected mice that were injected with KA on day 7 to induce seizures and exhibited the most severe neuronal ferroptosis. Brain tissue was collected, and 10 μm frozen brain sections were prepared. The neuronal changes in the CA1 and CA3 regions of the hippocampal tissue in epileptic mice after KA injection were analyzed using Nissl and silver staining. From the silver staining, we observed that the neuronal cell bodies in the CA1 and CA3 regions of the hippocampus in the TLE group mice appeared blurry, with shorter axons and a significant reduction in the number of neurons compared with the control group (Figure 3A). We used Nissl staining to observe hippocampal neurons. Compared with the control group, there was a significant reduction in the number of neurons in the CA1 and CA3 regions of the hippocampus on day 7 of experiencing seizures (Figure 3B).

### 2.4 Inhibition of FtMt exacerbated the imbalance of iron transport proteins and iron deposition induced during epilepsy

Animals were randomly divided into five groups according to the experimental requirements: normal mice (Control group), sham operation mice (Sham group), epileptic mice on day 7 after KA injection (7 d‐KA group), epileptic mice on day 7 after rAAV‐U6‐shRNA (scramble)‐CMV‐EGFP‐pA virus injection (Con‐KD group), and epileptic mice on day 7 after rAAV‐U6‐shRNA (*FtMt*)‐CMV‐EGFP‐pA virus injection (*FtM*t‐AAV‐KD group).

**FIGURE 4:**
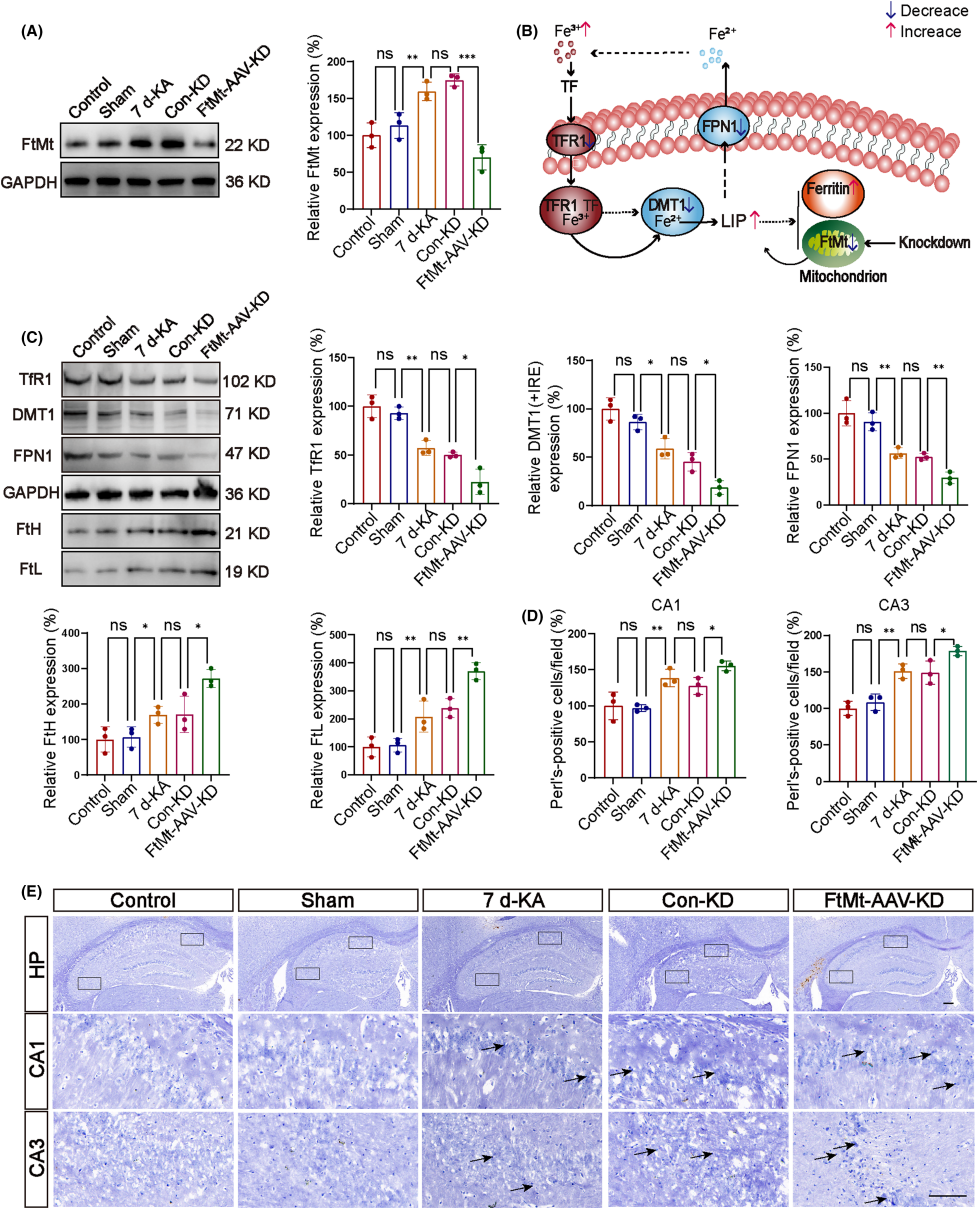
The impact of FtMt on the level of iron‐related transport proteins. (A) After inducing epilepsy, western blot analysis showed that the protein levels of FtMt in the hippocampus were decreased in the *FtM*t‐AAV‐KD group (*n* = 3 in each group). (B) Mechanistic diagram of iron ion metabolism. (C) After inducing epilepsy, western blot analysis showed a decrease in the protein levels of transferrin receptor 1 (TfR1), divalent metal transporter 1 (DMT1), and ferroportin 1 (FPN1) in the hippocampus, which was further decreased in the *FtMt*‐AAV‐KD group. Conversely, the protein levels of FtH and FtL in the hippocampus increased after inducing epilepsy, and *FtH* and FtL further increased in the *FtMt*‐AAV‐KD group (*n* = 3 in each group). (D, E) Perl's staining was used to measure Fe^3+^ in the hippocampal CA1 and CA3 regions. Fe^3+^ levels increased in epileptic mice, and the increase was more pronounced in the *FtMt*‐AAV‐KD group than the Con‐KD group (scale bar = 100 μm) (*n* = 3 in each group). Arrows point to Fe^3+^. HP stands for hippocampus. All data are depicted with SEM. *p < 0.05 **p < 0.01, ***p < 0.001, ns not significant.

After knocking down FtMt in the hippocampus, we studied the levels of iron homeostasis‐related proteins in the hippocampus of KA‐induced epileptic mice (Figure 4A). The mechanism diagram shows iron metabolism (Figure 4B). Then, we measured the expression of proteins involved in iron metabolism. Compared with the Con‐KD group, the levels of divalent metal transporter 1 (DMT1 + IRE), transferrin receptor 1 (TfR1), and ferroportin 1 (FPN1), which pumps iron ions out of the cell, were decreased in the *FtMt*‐AAV‐KD group. In contrast, the expression levels of FtH and FtL were increased in the *FtMt*‐AAV‐KD group (Figure 4C). Those results suggested that iron homeostasis was impaired in FtMt knockdown mice compared with the Con‐KD group.

We performed Fe^2+^ and Fe^3+^ staining in the hippocampal CA1 and CA3 regions using Lillie's Ferric Iron Stain kit and Prussian Blue Iron Stain Kit. Compared with the Con‐KD group, the levels of Fe^2+^ and Fe^3+^ were elevated in the *FtMt*‐AAV‐KD group (Figure 4D,E; Figure S4B). Those data suggest that the elevated iron ion concentration may be related to a decrease in the release of iron ions from cells due to the downregulation of proteins involved in iron homeostasis and the elevated ROS.

### 2.5 FtMt modulates neuronal ferroptosis and affects neuronal death caused by epilepsy

**FIGURE 5:**
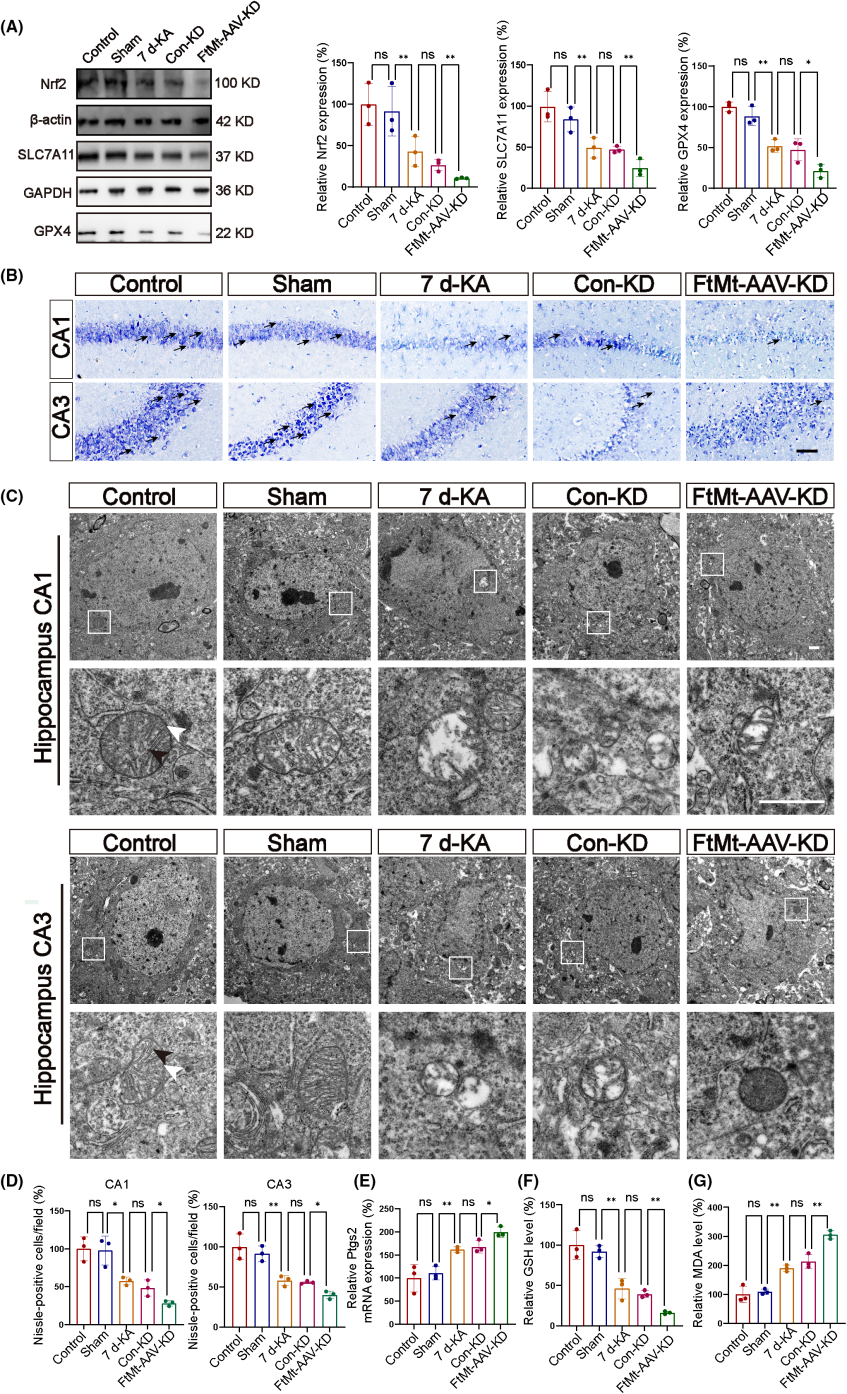
The expression of ferroptosis‐related indicators and neuronal damage in the hippocampal tissue of mice is influenced by FtMt. (A) The protein levels of GPX4, Nrf2, and SLC7A11 in hippocampal cells were decreased after status epilepticus and further decreased in the *FtMt*‐AAV‐KD group (*n* = 3 in each group). (B, D) FtMt affects neuronal death after status epilepticus. Representative image of Nissl staining in the hippocampus when seizures in a mouse model of seizures after FtMt knockdown (scale bar = 100 μm) (*n* = 3 in each group). Arrows point to Nissl‐positive cells. (C) Representative morphological changes to the mitochondria in different groups. Black arrows point to Mitochondrial crest. White arrows point to the Mitochondrial bilayer membrane (scale bar = 1 μm). (E–G) FtMt affects the expression of *Ptgs2* mRNA and the levels of GSH and MDA protein in the hippocampal tissue of mice (*n* = 3 in each group). All data are depicted with SEM. *p < 0.05 **p < 0.01, ***p < 0.001, ns not significant.

We investigated the changes in lipid peroxidation using commercial kits by measuring MDA and GSH levels. The 7 d‐KA group had markedly elevated MDA levels and markedly reduced GSH levels compared with the Control group (Figure 5F,G). Compared with the Con‐KD group, knockdown of the *FtMt* gene in the *FtMt*‐AAV‐KD group aggravated the increase in MDA and the decrease in GSH. In addition, we also assessed the mRNA level of *Ptgs2*. Compared with the Control group, the 7 d‐KA group exhibited significantly higher levels of *Ptgs2* mRNA. Furthermore, in the *FtMt*‐AAV‐KD group, there was a more pronounced increase in *Ptgs2* level compared with the Con‐KD group (Figure 5E). To measure changes in protein expression in the Nrf2/GPX4/SLC7A11 pathway, western blotting was also performed. There was a marked decrease in the levels of expression of Nrf2, SLC7A11, and GPX4 in the hippocampal tissues of the 7 d‐KA group compared with the Control group. Furthermore, in the *FtMt*‐AAV‐KD group, the expression of those proteins in the hippocampal tissues was significantly reduced compared with the 7 d‐KA group. No significant changes were observed in the Control group compared with the Sham group or in the Con‐KD group compared with the *FtMt*‐AAV‐KD group (Figure 5A). Those results indicated that knocking down FtMt may alleviate KA‐induced seizures through the Nrf2/SLC7A11/GPX4 pathway, which is involved in ferroptosis.

We also observed the different morphologies of the mitochondria in each group of hippocampal neuronal cells using transmission electron microscopy (TEM). Mitochondria in the 7 d‐KA group showed significant atrophy, rupture of the mitochondrial double membrane, and disappearance of the mitochondrial cristae compared with the control group. The *FtMt*‐AAV‐KD group exhibited more pronounced mitochondrial damage than the Con‐KD group. There were no significant differences in mitochondrial morphology between the Control group and the Sham group or between the 7 d‐KA group and the Con‐KD group (Figure 5C).

To study the effect of FtMt on neuronal survival, we observed hippocampal neurons using Nissl staining. Compared with the control group, the CA1 and CA3 regions were reduced in the 7 d‐KA group. Moreover, the *FtMt*‐AAV‐KD group showed a further reduction than the Con‐KD group (Figure 5B,D). Furthermore, animals were randomly divided into five groups according to the experimental requirements: normal mice (Control group), sham operation mice (Sham group), epileptic mice on day 7 after KA injection (7 d‐KA group), epileptic mice on day 7 after pcAAV‐CMV‐EGFP‐3xFLAG‐wpre virus injection (Con‐OE group), and epileptic mice on day 7 after pcAAV‐CMV‐ *FtM*t‐linker‐EGFP‐3xFLAG‐WPRE virus injection (*FtM*t‐AAV‐OE group). Compared with the Con‐OE group, the CA1 and CA3 regions were increased in the *FtM*t‐AAV‐OE group (Figure 7E,G).

Those results indicated that FtMt could modulate ferroptosis in a mouse model of KA‐induced seizures.

### 2.6 Inhibition of FtMt increased glutamate‐induced ROS in primary mouse hippocampal neuronal cells

**FIGURE 6:**
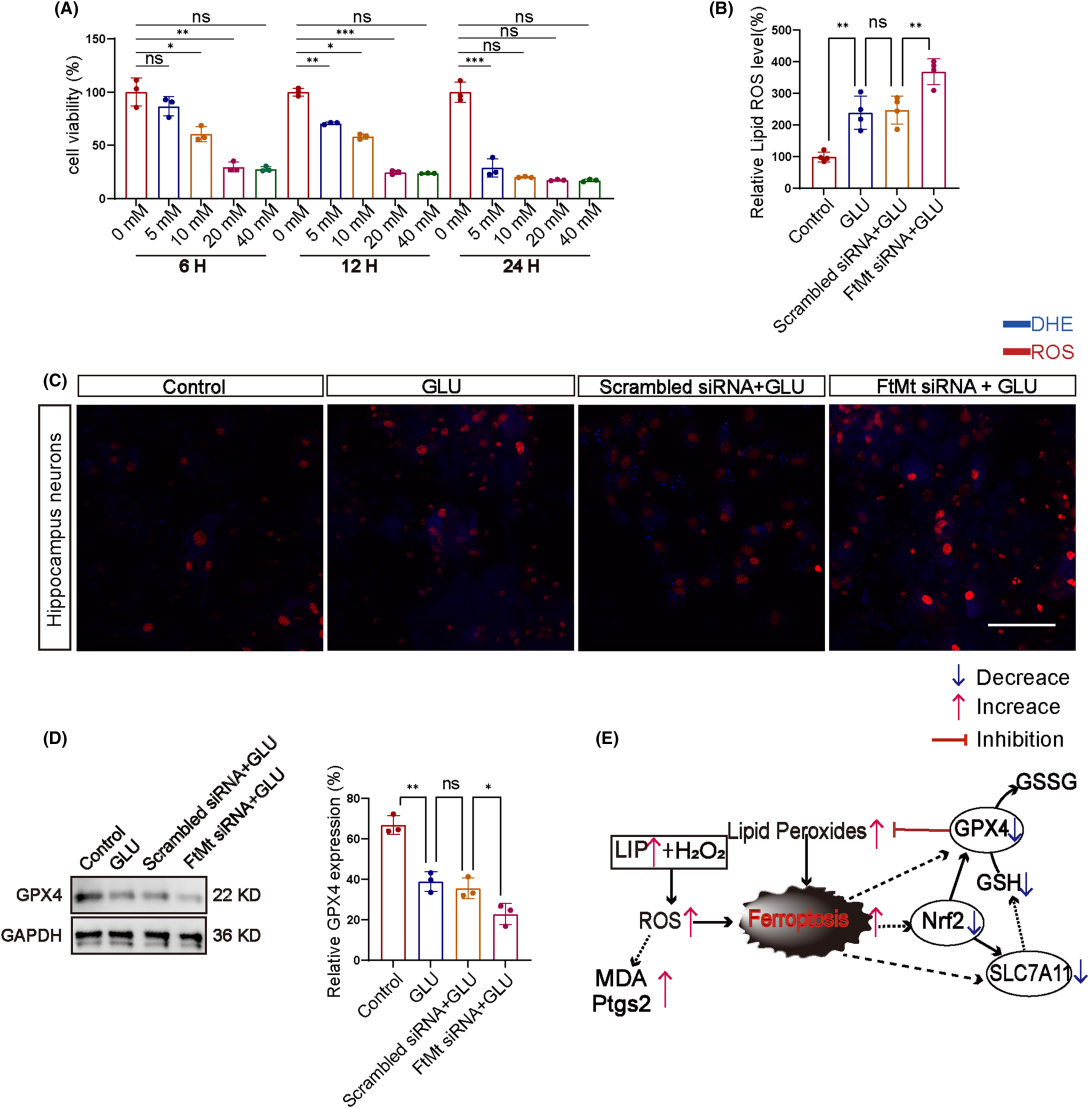
Effects of glutamate on primary mice hippocampal neuronal cells. (A) Glutamate decreases primary mice hippocampal neuronal cell viability in a dose‐ and time‐dependent manner. (B, C) The effects of glutamate intervention on primary mouse hippocampal neurons and the influence of FtMt on their ROS levels (*n* = 4 in each group) (scale bar = 100 μm). (D) Western blot assay was used to detect the expression of GPX4 (*n* = 3 in each group). (E) Schematic diagram of metabolism of related ferroptosis pathways. All data are depicted with SEM. *p < 0.05, **p < 0.01, ***p < 0.001, ns not significant.

We demonstrate the process of extraction and identification of primary hippocampal neurons using a schematic diagram (Figure S3A,B). We investigated the effect of ROS on neuronal cell death induced by glutamate in primary mouse hippocampal neurons and the relationship between ROS and FtMt. The CCK‐8 assay was used to assess the viability of primary mouse hippocampal neuronal cells exposed to different concentrations of glutamate at different times. The cell culture was treated with glutamate at concentrations of 5, 10, 20, and 40 mM to induce cell death, and the CCK‐8 assay was performed at 6, 12, and 24 h. The average survival capacity of primary hippocampal neurons significantly decreased with increasing glutamate concentrations and a prolonged latency period. Treatment with 20 mM glutamate salt for 12 h induced close to 50% cell death (Figure 6A).

FtMt was knocked down in primary mouse hippocampal neurons using siRNA transfection. According to the experimental requirements, the cells were divided into four groups: cells cultured in normal culture medium (Control group), cells cultured in 20 mM glutamate medium (GLU group), cells cultured in 20 mM glutamate medium with scrambled siRNA (Scrambled siRNA group), and cells cultured in 20 mM glutamate medium with *FtMt* siRNA (*FtMt* siRNA group). Those groups were then compared with analyze the changes in MDA and GSH between the *FtMt* siRNA group and the Scrambled group. The Scrambled siRNA group and *FtMt* siRNA group were added to the culture medium for 12 h, along with 20 mM glutamate, to study the function of FtMt in regulating neuronal ROS in an in vitro model of glutamate‐induced epilepsy. Intracellular ROS were measured using the fluorescent probe, DHE. Compared with the control group, the level of red fluorescence, which is representative of ROS, was significantly increased in glutamate‐treated hippocampal neurons. Compared with the scrambled siRNA group, treatment with *FtMt* siRNA group further enhanced the levels of red fluorescence in glutamate‐treated primary mice hippocampal neurons, effectively increasing the levels of ROS induced by glutamate (Figure 6B,C).

In addition, we detected the protein expression of GPX4 in different groups by Western blot method, and the results showed that compared with the control group, the GLU and Scrambled siRNA + GLU groups had a significant decrease in GPX4. In contrast, the FtMt siRNA + GLU group had the most severe reduction (Figure 6D). We use the mechanism diagram to show the pathway mechanism (Figure 6E).

### 2.7 Changes in FtMt expression in mouse hippocampal tissues modulate seizure activity in mice

**FIGURE 7:**
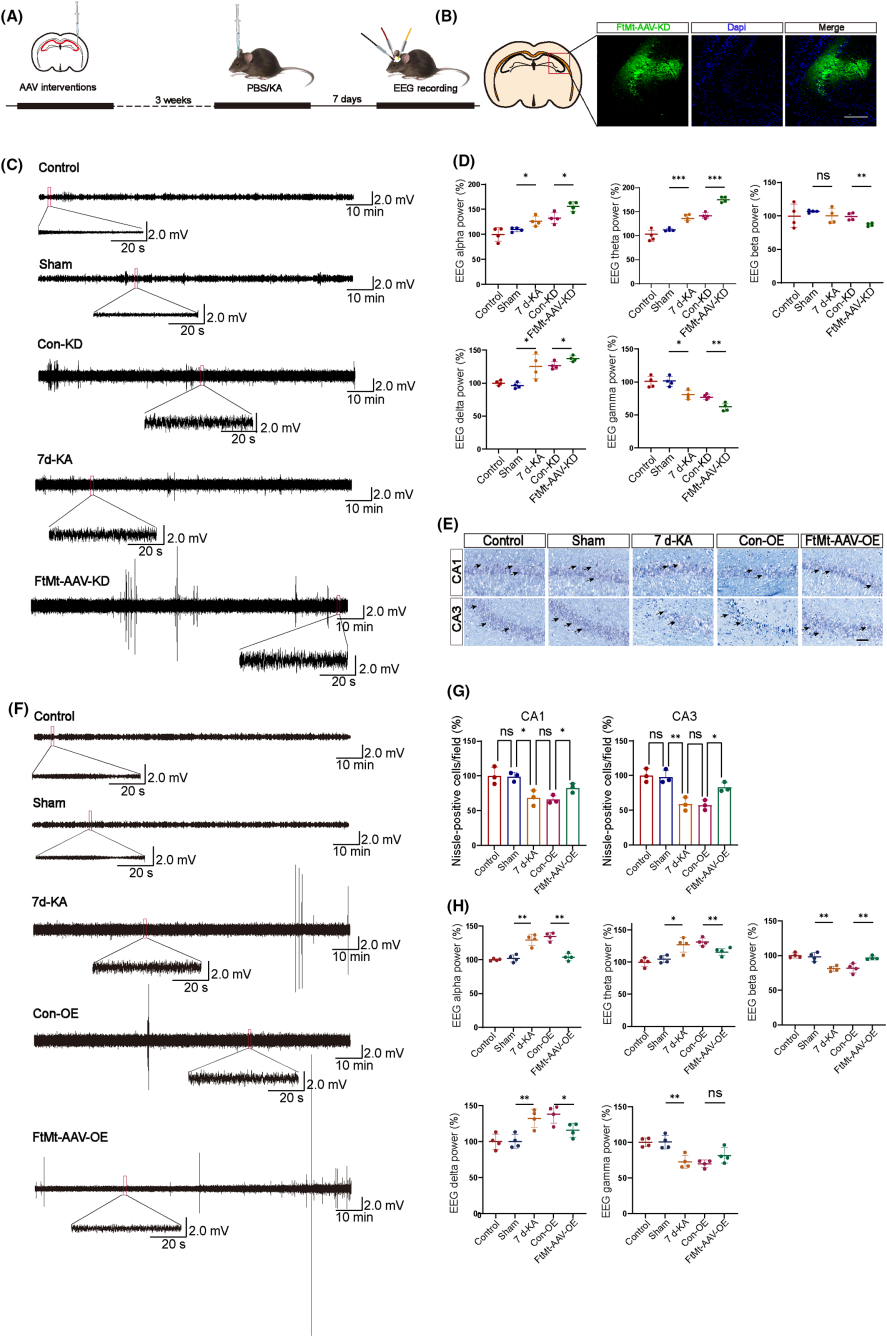
Changes in FtMt in the hippocampus of epileptic mice affect their brain electrical activity. (A) Graphical representation of the experimental timeline. (B) Immunofluorescence plots of AAV virus expression (scale bar = 100 μm). (C, D, F, H) Effect of FtMt expression changes in the mouse hippocampus on alpha, theta, beta, delta, and gamma waves (*n* = 4 in each group). (E, G) Representative image of Nissl staining in the hippocampus when seizures in a mouse model of seizures after FtMt overexpression (scale bar = 100 μm) (*n* = 3 in each group). Arrows point to Nissl‐positive cells. All data are depicted with SEM. *p < 0.05, **p < 0.01, ***p < 0.001, ns not significant.

We injected an *FtMt* knockdown virus into the CA3 region of the mouse hippocampus to investigate the regulatory role of FtMt in modulating seizure activity in the KA‐induced TLE mouse model (Figure 7A,B). Compared with the Con‐KD group, the *FtMt*‐AAV‐KD group exhibited increased alpha (8–12 Hz) and theta (4–8 Hz) waves in the brain EEG, with the most significant increase observed in the theta waves. However, compared with the Con‐KD group, the *FtMt*‐AAV‐KD group showed a decrease in beta (12–30 Hz), delta (0.5–4 Hz), and gamma (30–100 Hz) waves in the brain EEG. The 7 d‐KA group of the beta wave did not change compared with the Control group. A significant change in the power spectrum of the FtMt‐AAV‐KD group compared with the Con‐KD group was found by power spectrum analysis (Figure 7C,D; Figure S4A).

We then injected the *FtMt* overexpressing virus into the CA3 region of the mouse hippocampus. The results showed that the *FtMt*‐AAV‐OE group exhibited reduced α (8–12 Hz), theta (4–8 Hz) waves, and delta (0.5–4 Hz) on EEG compared with the Con‐OE group. However, the *FtMt*‐AAV‐OE group showed an increase in β waves (12–30 Hz) on EEG compared with the Con‐OE group, while gamma waves (30–100 Hz) were not statistically significant (Figure 7F,H).

### 2.8 Elevated expression of Nrf2 in epileptic mice affects the expression of FtMt

To explore the molecular mechanisms of Nrf2 in KA‐induced epilepsy, we investigated the effects of Nrf2 on FtMt expression using adenoviral overexpression.

The animals were randomly divided into five groups according to the experimental requirements: normal mice (Control group), sham operation mice (Sham group), epileptic mice on day 7 after KA injection (7 d‐KA group), epileptic mice on day 7 after 3 weeks of pAAV‐CMV‐MCS‐3xFLAG‐WPRE virus injection (Con‐OE group), and epileptic mice on day 7 after 3 weeks of pAAV‐CMV‐Nfe212‐3xFLAG‐WPRE virus injection (*Nrf2*‐AAV‐OE group).

**FIGURE 8:**
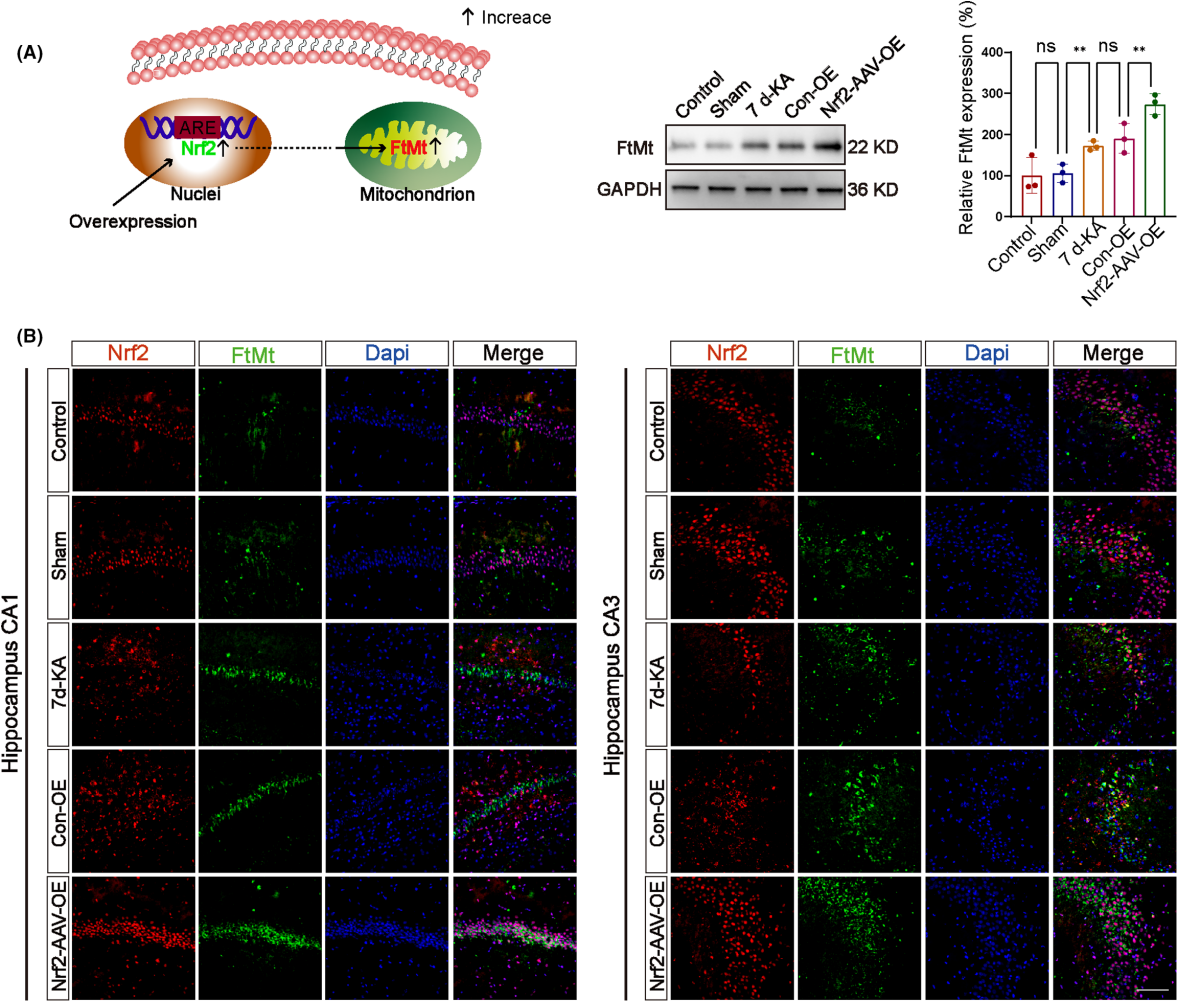
Nrf2 affects the expression of FtMt in the hippocampus of epileptic mice. (A) Schematic diagram of the relationship between Nrf2 and FtMt mechanism. (B) Western blot analysis showed an increase in the protein levels of FtMt in the hippocampus, which further increased in *Nrf2*‐AAV‐OE group (*n* = 3 in each group). (C) Immunofluorescence staining revealed an increase in the expression of FtMt in the CA1 and CA3 regions of the hippocampi in epileptic mice, with further enhancement in the *Nrf2*‐AAV‐OE group (scale bar = 100 μm). All data are depicted with SEM. *p < 0.05, **p < 0.01, ***p < 0.001, ns not significant.

After enhancing the expression of Nrf2 in the hippocampus of mice, we utilized protein blot analysis to investigate the expression of FtMt in the hippocampus of mice in the KA‐induced Con‐OE group and *Nrf2*‐AAV‐OE group (Figure 8A). Compared with the Con‐OE group, the protein expression level of FtMt was increased in the *Nrf2*‐AAV‐OE group. After overexpressing Nrf2 in the hippocampus of epileptic mice, we investigated the level of FtMt protein in the hippocampus of KA‐induced epileptic mice using immunofluorescence analysis (Figure 8B). In the CA1 and CA3 regions of mouse hippocampal neurons, a significant increase in Nrf2 and FtMt levels was observed in the *Nrf2*‐AAV‐OE group compared with the Con‐KD group. Thus, Nrf2 may regulate FtMt in neuronal cells to influence iron‐induced neuronal cell death and iron homeostasis during epilepsy.

## 3 DISCUSSION

In this study, we have demonstrated that the level of FtMt protein in the hippocampus of KA‐induced TLE mice was significantly increased on day 7, and the ferroptosis‐related indicators were most seriously affected. By reducing the level of FtMt protein expression, we observed an impact on the level of iron homeostasis‐related proteins in the hippocampus of mice with epilepsy. The imbalance in iron homeostasis exacerbates the level of iron‐mediated cell death, leading to the aggravation of epilepsy. The decrease in FtMt expression also increased the accumulation of ROS in a glutamate‐induced primary mouse hippocampal neuronal cell culture model. In addition, we showed for the first time that there may be a relationship between Nrf2 and FtMt expression. By increasing the expression of Nrf2 in the hippocampus of KA‐induced epileptic mice, the expression of FtMt protein also increased.

Epilepsy is a common brain disease that is characterized by repetitive and spontaneous seizures.^29^ Ferroptosis involves three main factors: deficiency of the GSH/*GPX4* redox system Xc, abnormal iron ion metabolism, and aberrant lipid peroxidation.^6^, ^30^, ^31^, ^32^ Our research found that GPX4 expression in the hippocampus of epileptic mice was most significantly reduced on day 7. Earlier studies linked lipid peroxidation and iron accumulation to the onset of several neurological conditions as well as reduced levels of GPX4 and GSH.^33^ Animal studies also showed that seizures can cause ptosis and are associated with a significant decrease in GPX4 expression.^14^, ^34^ Based on that finding, we speculate that the decreased expression of GPX4 during epilepsy may exacerbate the accumulation of intracellular free iron ions, thereby affecting the level of expression of iron metabolism‐related proteins.

The expression of FtH and FtL was most significantly increased in the hippocampus of mice with epilepsy on day 7. Previous studies showed that FtH and FtL can bind to excess free iron ions in the cytoplasm of cells to regulate ferroptosis.^35^ Therefore, we believe epilepsy can be treated by improving ferroptosis by chelating excessive free iron ions in the cytoplasm.

Previous studies have found that FtMt can chelate intracellular free iron. Abnormalities in FtMt are associated with mitochondrial disease and neurodegenerative disease.^17^, ^34^ We discovered that FtMt can alleviate neuronal cell damage by regulating iron metabolism in the hippocampus tissue of epileptic mice. At the same time, FtL and FtH, as members of the iron transport family, are also involved in the regulation of oxidative stress and neuronal ferroptosis in epilepsy, despite being located in different intracellular locations from FtMt. Previous studies have confirmed the structural and functional relationship between FtMt and FtH/FtL. In our research, FtMt can affect FtL and FtH and participate in the homeostatic regulation of intracellular iron ions. Knockdown of FtMt increases the free iron ions in cells, which in turn leads to a further increase in the compensatory properties of FtL and FtH. These proteins protect cells from ferroptosis caused by oxidative stress by regulating the storage and transport of iron ions, reducing the chance of iron ions participating in oxidative stress. However, the specific mechanisms and interactions still need further research to be further studied.^17^, ^34^

Iron ions participate in maintaining normal human body functions in the forms of Fe^2+^ and Fe^3+^.^36^ Our study found that decreasing the expression of FtMt led to an increase in the level of ferritin, including FtH and FtL. The reduced function of FtMt also increased intracellular Fe^2+^ and Fe^3+^. Based on those findings, we speculate that FtMt may chelate free iron ions by increasing its expression in hippocampal tissue. By reducing FtMt expression, there is a decrease in the binding of Fe^2+^ to FtMt within the mitochondria, leading to an increase in Fe^2+^ in the cytoplasm. This results in FtH and FtL to sequester the excessive free iron ions. Following a decrease in FtMt expression, the expression of FPN1 is reduced. Previous studies have demonstrated that FPN1 is the only efflux channel for Fe^2+^.^37^ Therefore, we speculate that FtMt may influence the expression of FPN1, leading to the accumulation of free iron ions in the cytoplasm.

Additionally, previous research has shown that DMT1 can convert intracellular Fe^3+^ to Fe^2+^.^38^ We found that the expression of DMT1 decreases following the reduction of FtMt expression. This may be due to the involvement of FtMt in the expression of DMT1, resulting in a reduction in the conversion of Fe^3+^ to Fe^2+^. Therefore, we further examined the changes in gene expression related to ferroptosis in the hippocampi and primary hippocampal neurons of epileptic mice. The most significant changes in ferroptosis biomarkers occurred in the hippocampus of mice 7 days after epileptic seizures. The expression levels of SLC7A11, Nrf2, and GSH decreased, while ROS accumulation increased in the CA1 and CA3 regions of the hippocampus. Those findings were consistent with previous studies.^21^, ^39^

To investigate the impact of FtMt on ferroptosis‐related pathways, we knocked down the expression of FtMt in the hippocampus of epileptic mice. The expression of genes associated with ferroptosis further deteriorated, mitochondrial shrinkage increased, mitochondrial cristae decreased, double membrane rupture occurred, and intracellular ROS levels increased. These findings suggest a potential mechanism by which FtMt alleviates iron‐mediated cell death in KA‐induced epilepsy. FtMt can sequester excess free iron ions and prevent their accumulation in mitochondria. Additionally, FtMt regulates the excess of free iron ions by influencing the expression of iron homeostasis‐related proteins, thus, avoiding the generation of excessive ROS within cells. The protective role of FtMt helps reduce lipid peroxidation and cell membrane damage, ultimately regulating neuronal ferroptosis associated with epilepsy.

Electrophysiological testing revealed that after FtMt knockdown, the brain electrical amplitude and frequency increased in epileptic mice. However, the specific mechanisms of epilepsy are currently unknown. Modulating iron homeostasis through FtMt may represent a novel therapeutic approach for epilepsy.

Nrf2 is a transcription factor that can regulate cell ferroptosis through the antioxidant stress response.^25^ Some studies have shown that Nrf2 can regulate FtH and FtL in brain injury models.^40^ The impact of Nrf2 on FtH and FtL may involve FtMt, but previous studies on TBI did not investigate the expression of FtMt. Therefore, we conducted a study investigating the relationship between Nrf2 and FtMt in epilepsy. We demonstrated the association of Nrf2 with FtMt in epileptic mice and found that the overexpression of Nrf2 in the hippocampus of epileptic mice enhanced the expression of FtMt. We concluded that Nrf2 regulates ferroptosis during epilepsy through the expression of FtMt. Additionally, the mechanism by which Nrf2 affects ferroptosis through its involvement in the regulation of FtMt remains unknown.

In summary, our data demonstrate the role and regulatory mechanism of FtMt in improving epilepsy and its associated ferroptosis through its impact on iron ion metabolism. Additionally, the changes in Nrf2 expression during epilepsy can influence the expression of FtMt. Ultimately, this research may provide new strategies for the treatment of epilepsy.

## 4 MATERIALS AND METHODS

### 4.1 Animals and animal model

Adult male mice (C57BL/6 strain, 25–30 g) were used in the experiment. All mice were housed under a standard 12 h light/dark cycle with a controlled temperature (22°C) at the Experimental Animal Center, Zhujiang Hospital, Southern Medical University, China. Food and water were provided ad libitum. All procedures were approved by the Animal Ethics Committee of Zhujiang Hospital, Southern Medical University, and conducted following international standards (certificate number: LAEC‐2022‐198).

The mouse model of KA‐induced epilepsy has been widely reported.^41^ The mouse model of KA‐induced epilepsy was established 21 days after injection of AAV. The mice were anesthetized with pentobarbital sodium (50 mg/kg) via intraperitoneal injection and then secured in a stereotaxic apparatus (RWD Life Science Co., Ltd., Shenzhen, China) for KA injection. Using a 0.5 μL microsyringe (Hamilton, Reno, NV, USA), 1.0 nmol of KA (Sigma‐Aldrich Co., St. Louis, USA) was dissolved in 0.05 μL of physiological saline. The dissolved KA was injected into the right hippocampus based on the mouse brain in stereotaxic coordinates (George Paxinos, 2001, The Mouse Brain in Stereotaxic Coordinates, USA, Academic Press, 350 pp.) to establish an epilepsy model. The injection coordinates were AP: −2.0 mm, ML: −2.5 mm, DV: −2.2 mm. To ensure proper absorption of the drug and prevent reflux along the injection tract, the syringe was left within the hippocampus for 5 min after injection, maintaining a total injection time of 3 min. Mice injected with KA by stereotactic placement are placed under a video surveillance system to record seizures at 3, 7, 14, and 28 days. The seizure grades recorded according to the Racine scale were as follows: stage 0, no response or behavior arrest; stage 1, chewing or facial twitches; stage 2, chewing and head nodding or wet dog shakes; stage 3, unilateral forelimb clonus; stage 4, bilateral forelimb clonus and rearing; stage 5, bilateral forelimb clonus, rearing and falling.^42^

rAAV‐U6‐shRNA (scramble)‐CMV‐EGFP‐pA and rAAV‐U6‐shRNA (*FtMt*)‐CMV‐EGFP‐pA were designed and procured from Wuhan BrainVTA. pAAV‐CMV‐MCS‐3xFLAG‐WPRE and pAAV‐CMV‐Nfe212‐3xFLAG‐WPRE were designed and procured from Shanghai OBiO. pcAAV‐CMV‐EGFP‐3xFLAG‐WPRE and pcAAV‐CMV‐ *FtM*t‐linker‐EGFP‐3xFLAG‐WPRE were designed and procured from Shanghai OBiO.

### 4.2 EEG recording

Following hippocampal injections, we selected four mice from each group for brain map recording. Following anesthesia with pentobarbital (50 mg/kg), mice had three metal electrodes implanted into their skull to record the EEGs. The recording electrodes were placed on the contralateral hippocampus. The reference electrode and ground electrode were placed on the frontal bone and occipital bone, respectively. The EEG signals were recorded using an EEG recorder (Solar, Beijing, China). As described previously, the injection site was located in the right hippocampus. The average power within six frequency bins between 0 and 100 Hz was calculated for each 1‐min EEG segment. These frequency bins included delta (0.5–4 Hz), theta (4–8 Hz), alpha (8–12 Hz), beta (12–30 Hz), and gamma (30–100 Hz).^43^, ^44^

### 4.3 Primary hippocampal neuron extraction

The embryonic hippocampal tissue was digested in a buffer solution (2 mg/mL papain, Worthington, USA) in Dulbecco's modified Eagle's medium, for 20 min. After digestion, the tissue was collected in a tissue culture dish containing 10% heat‐inactivated fetal bovine serum, 1% antibiotics‐antimycotics, and 2 mM L‐glutamine (Gibco, CA, USA). Half of the culture medium was replaced every 2 days.

### 4.4 CCK8 assay

On day 7 of culturing primary neuronal cells (1 × 10^4^ cells/well) in a 96‐well plate, the cells were treated with glutamate at concentrations of 5, 10, 20, and 40 mM for 6, 12, and 24 h. Cell viability was determined using the CCK‐8 assay (Dojindo, Kumamoto, Japan). Primary hippocampal neurons (5 × 10^5^ cells/well) were then transferred to a six‐well plate and grown for 7 days.

### 4.5 Cell transfection

Following the manufacturer's instructions, cells were transfected with siRNA using Lipofectamine 2000 (Invitrogen, CA, USA). After overnight transfection with siRNA, glutamate was added at a concentration of 20 mM. Primary hippocampal neurons were randomly divided into four groups: cells without treatment (Control group), cells with glutamate treatment group, cells with scrambled siRNA and glutamate treatment (Scrambled siRNA group), and cells with *FtMt* siRNA and glutamate treatment (*FtMt* siRNA group).

### 4.6 Immunofluorescence staining

Immunofluorescence methods have been widely described.^41^ Briefly, 10 μm thick tissue slices were fixed in methanol for 30 min, washed three times in phosphate‐buffered saline (PBS), cleaved with 0.3% Triton X‐100 for 5 min, and mounted in 10% donkey serum working solution (Boster Biological Technology, Wuhan, China) for 60 min. The sliced and mixed samples were incubated with primary antibodies for 12 h at 4°C. Afterward, the samples were washed with PBST to remove residual antibodies and then incubated with the second antibody for 1 h at room temperature in the dark. Primary mouse hippocampal neurons were stained using the ROS red fluorescence probe kit (Biobraille, Shandong, China). The primary antibodies included rabbit anti‐FTMT (1:200; Abcam, USA), mouse anti‐NEUN antibodies (1:100, Abcam, MA, USA), and rabbit anti‐NRF2 (1:300; Affinity, Shanghai, China). Mouse anti‐IBA1 antibodies (1:100; Abcam, MA, USA) and mouse anti‐GFAP antibodies (1:100; Abcam, MA, USA) were used to identify the expression location of *FtMt*. The secondary antibodies used were donkey anti‐mouse Alexa Fluor 650 (1:100, Boster Biological Technology, Wuhan, China), donkey anti‐rabbit Alexa Fluor 488 (1:100, Boster Biological Technology), and double‐fluorescence immunohistochemical mouse/rabbit kit (ImmunoWay, Beijing, China).

### 4.7 MDA and GSH level measurements

After intraperitoneal injection in mice, the mice were anesthetized with pentobarbital sodium (50 mg/kg), and the hippocampal tissue was collected after perfusion with physiological saline through the apex of the heart. Following the manufacturer's instructions, use the corresponding commercial assay kits (GSH, MDA; Beyotime Technology, Shanghai, China) to measure the levels of metabolic products GSH and MDA associated with ferroptosis.

### 4.8 Western blot

We collected the right hippocampal tissue samples from different groups of mice for protein immunoblot analysis. We separated the extracted proteins from mouse hippocampal tissue using 10%–15% SDS‐polyacrylamide gel electrophoresis (based on the different molecular weights of the proteins). The proteins were then transferred to 0.22 μm polyvinylidene fluoride (PVDF) membranes (Millipore, MA, USA).

The membrane was then immersed in 5% skim milk at room temperature for 60 min, and the strips were incubated with rabbit anti‐FtMt (1:1000; Thermo Fisher, MA, USA), rabbit anti‐FtL (1:1000; Abcam, MA, USA), rabbit anti‐FtH (1:1000; Abcam, MA, USA), rabbit anti‐FPN1 (1:1000; Proteintech, Wuhan, China), rabbit anti‐GPX4 (1:1000; Abcam, MA, USA), rabbit anti‐SLC7A11 (1:1000; Abcam, MA, USA), rabbit anti‐DMT1(+IRE) (1:1000; ImmunoWay, Beijing, China), rabbit anti‐TFR1 (1:1000; Abcam, MA, USA), and rabbit anti‐Nrf2 (1:1000; Affinity, Shanghai, China) primary antibodies. After washing with TBST, the membranes were incubated with horseradish peroxidase (HRP)‐conjugated secondary antibodies (anti‐mouse or anti‐rabbit secondary antibodies were used (1:5000; Proteintech, Wuhan, China)) for 60 min. Subsequently, the residual antibodies were removed with TBST and visualized using an enhanced chemiluminescence system (Advansta, CA, USA). The protein bands were then analyzed for relative protein levels using ImageJ software.

### 4.9 RNA isolation and quantitative PCR

The quantitative real‐time reverse transcription polymerase chain reaction (qRT‐PCR) measured ferroptosis‐associated *Ptgs2* mRNA levels. Total RNA was obtained from hippocampal tissues using TRIzol reagent (Invitrogen, MA, USA), according to the manufacturer's instructions. Subsequently, the total RNA from the samples was reverse transcribed into cDNA using the PrimeScript™ RT Kit (Takara, Shiga, Japan). Amplification was performed using SYBR Green PCR Master Mix (GenStar, Beijing, China) on the Bio‐Rad CFX Connect system. The detailed primer sequences are shown in (Figure S5).

### 4.10 TEM

Fresh hippocampus were soaked electron microscopy fixative (Solarbio, Beijing, China) overnight at 4°C, followed by a 30‐min immersion in 1% osmic acid in 0.1 M phosphate buffer. The tissues were then dehydrated and embedded in Araldite using a series of graded ethanol and acetone. Ultrathin sections (Lecia, Wetzlar, Germany) were cut using an ultramicrotome and stained with uranyl acetate and lead citrate. Examination was performed using an electron microscope (Hitachi, Tokyo, Japan).

### 4.11 Nissl staining

After anesthetizing the mice with sodium pentobarbital, they were fixed by perfusion through the left ventricle with pre‐chilled physiological saline and 4% paraformaldehyde. The brains were dehydrated in a 40% sucrose solution for 48 h and then fixed in 4% paraformaldehyde. The brains were sliced into 10 μm thick sections and immersed in Nissl staining solution. Following Nissl staining, sections were washed twice with distilled water for 2 s each time. They were then immersed in 95% ethanol for approximately 3–5 s, followed by immersion in absolute ethanol for 1 min. The sections were subsequently air‐dried, cleared, and covered with a coverslip. The surviving neurons in the hippocampal CA1 and CA3 regions were analyzed in the stained sections, and the number of viable neurons was determined by assessing the changes in Nissl bodies and nucleoli.

### 4.12 Perl's staining and Lillie staining

The levels of Fe^2+^ and Fe^3+^ were evaluated using Lillie's trivalent iron staining kit (Solarbio, Beijing, China) and Perl's staining kit (Solarbio) enhanced with 3,3‐diaminobenzidine tetrahydrochloride (DAB), following the manufacturer's instructions.

### 4.13 Silver staining

The silver staining method modified from Palmgren's silver impregnation staining method allows the visualization of alterations in neuronal fibers and synapses during illnesses.^42^, ^45^ Each mouse's paraffin‐embedded brain tissue sections were deparaffinized in xylene and absolute ethanol and then treated for 5 min in acidic formaldehyde before being incubated for 3–5 min in a glycine‐silver solution. After staining in a reducing solution, the sections were examined under an optical microscope. On a yellow background, axons, dendrites, collagen fibers in nerve cells, and neurofibrillary tangles (NFTs) were dyed dark brown or black.

### 4.14 Statistical analysis

Levene was used for homogeneity of variance. The Shapiro–Wilk test is used to verify that the data are normally distributed. For the comparison of normally distributed data, the unpaired *t*‐test, ANOVA, and then the Tukey test were performed as a post‐hoc analysis. To compare the nonnormally distributed data with the nonvariance homogeneity data, the Kruskal–Wallis test was performed. The above experiments were carried out according to the principles of randomization and blinding. All analyses were performed using GraphPad Prism version 9.5.0 software, and *p* < 0.05 was considered statistically significant. Data are presented as the mean ± SEM. The significance threshold is represented as *p < 0.05, **p < 0.01, and ***p < 0.001. Detailed information regarding statistical tests and results can be found in the figure legends.

## AUTHOR CONTRIBUTIONS

YS and MG performed experiments and contributed substantially to the analysis and interpretation of the data and reviewed the manuscript. YG, JZ, and BW reviewed the manuscript. XH and JZ contributed to this work with materials and methods. YS, MG, YG, BW, and ZY designed the experimental research, made substantial contributions to the analysis and interpretation of the data, drafted, and critically reviewed the manuscript. All authors read and approved the final manuscript.

## FUNDING INFORMATION

This study was supported by Grants from the National Natural Science Foundation of China (82071452, 82271488) and Guangdong Basic and Applied Basic Research Foundation (2022A1515140149).

## CONFLICT OF INTEREST STATEMENT

The authors declare that there is no conflict of interest in the publication of this article.

## Supporting information


Figures S1–S6

## Data Availability

All data are contained in the manuscript or in supplementary material provided by the authors.

## References

[1] Fisher RS , Acevedo C , Arzimanoglou A , et al. ILAE official report: a practical clinical definition of epilepsy. Epilepsia. 2014;55:475‐482. 24730690 10.1111/epi.12550

[2] Du K , He M , Zhao D , et al. Mechanism of cell death pathways in status epilepticus and related therapeutic agents. Biomed Pharmacother. 2022;149:112875. 35367755 10.1016/j.biopha.2022.112875

[3] Vezzani A , French J , Bartfai T , Baram TZ . The role of inflammation in epilepsy. Nat Rev Neurol. 2011;7:31‐40. 21135885 10.1038/nrneurol.2010.178 PMC3378051

[4] Dixon SJ , Lemberg KM , Lamprecht MR , et al. Ferroptosis: an iron‐dependent form of nonapoptotic cell death. Cell. 2012;149:1060‐1072. 22632970 10.1016/j.cell.2012.03.042 PMC3367386

[5] Cai Y , Yang Z . Ferroptosis and its role in epilepsy. Front Cell Neurosci. 2021;15:696889. 34335189 10.3389/fncel.2021.696889 PMC8319604

[6] Do Van B , Gouel F , Jonneaux A , et al. Ferroptosis, a newly characterized form of cell death in Parkinson's disease that is regulated by PKC. Neurobiol Dis. 2016;94:169‐178. 27189756 10.1016/j.nbd.2016.05.011

[7] Chen D , Fan Z , Rauh M , Buchfelder M , Eyupoglu IY , Savaskan N . ATF4 promotes angiogenesis and neuronal cell death and confers ferroptosis in a xCT‐dependent manner. Oncogene. 2017;36:5593‐5608. 28553953 10.1038/onc.2017.146 PMC5633655

[8] Alim I , Caulfield JT , Chen Y , et al. Selenium drives a transcriptional adaptive program to block Ferroptosis and treat stroke. Cell. 2019;177:1262‐1279.e25. 31056284 10.1016/j.cell.2019.03.032

[9] Xie BS , Wang YQ , Lin Y , et al. Inhibition of ferroptosis attenuates tissue damage and improves long‐term outcomes after traumatic brain injury in mice. CNS Neurosci Ther. 2019;25:465‐475. 30264934 10.1111/cns.13069 PMC6488926

[10] Ashraf A , Jeandriens J , Parkes HG , So PW . Iron dyshomeostasis, lipid peroxidation and perturbed expression of cystine/glutamate antiporter in Alzheimer's disease: evidence of ferroptosis. Redox Biol. 2020;32:101494. 32199332 10.1016/j.redox.2020.101494 PMC7083890

[11] Kumar A , Kumar V , Singh K , et al. Therapeutic advances for Huntington's disease. Brain Sci. 2020;10:43. 31940909 10.3390/brainsci10010043 PMC7016861

[12] Xie R , Zhao W , Lowe S , et al. Quercetin alleviates kainic acid‐induced seizure by inhibiting the Nrf2‐mediated ferroptosis pathway. Free Radic Biol Med. 2022;191:212‐226. 36087883 10.1016/j.freeradbiomed.2022.09.001

[13] Ye Q , Zeng C , Dong L , Wu Y , Huang Q , Wu Y . Inhibition of ferroptosis processes ameliorates cognitive impairment in kainic acid‐induced temporal lobe epilepsy in rats. Am J Transl Res. 2019;11:875‐884. 30899387 PMC6413264

[14] Mao X , Zhou H , Jin W . Ferroptosis induction in Pentylenetetrazole kindling and pilocarpine‐induced epileptic seizures in mice. Front Neurosci. 2019;13:721. 31379480 10.3389/fnins.2019.00721 PMC6652743

[15] Ye Q , Zeng C , Luo C , Wu Y . Ferrostatin‐1 mitigates cognitive impairment of epileptic rats by inhibiting P38 MAPK activation. Epilepsy Behav. 2020;103:106670. 31864943 10.1016/j.yebeh.2019.106670

[16] Chen K , Guan QW , Yin XX , Wang ZJ , Zhou HH , Mao XY . Ferrostatin‐1 obviates seizures and associated cognitive deficits in ferric chloride‐induced posttraumatic epilepsy via suppressing ferroptosis. Free Radic Biol Med. 2022;179:109‐118. 34952157 10.1016/j.freeradbiomed.2021.12.268

[17] Wang P , Cui Y , Ren Q , et al. Mitochondrial ferritin attenuates cerebral ischaemia/reperfusion injury by inhibiting ferroptosis. Cell Death Dis. 2021;12:447. 33953171 10.1038/s41419-021-03725-5 PMC8099895

[18] Drysdale J , Arosio P , Invernizzi R , et al. Mitochondrial ferritin: a new player in iron metabolism. Blood Cells Mol Dis. 2002;29:376‐383. 12547228 10.1006/bcmd.2002.0577

[19] Levi S , Corsi B , Bosisio M , et al. A human mitochondrial ferritin encoded by an Intronless gene. J Biol Chem. 2001;276:24437‐24440. 11323407 10.1074/jbc.C100141200

[20] Levi S , Arosio P . Mitochondrial ferritin. Int J Biochem Cell Biol. 2004;36:1887‐1889. 15203103 10.1016/j.biocel.2003.10.020

[21] Carmona‐Aparicio L , Pérez‐Cruz C , Zavala‐Tecuapetla C , et al. Overview of Nrf2 as therapeutic target in epilepsy. Int J Mol Sci. 2015;16:18348‐18367. 26262608 10.3390/ijms160818348 PMC4581249

[22] Bou‐Abdallah F , Santambrogio P , Levi S , Arosio P , Chasteen ND . Unique iron binding and oxidation properties of human mitochondrial ferritin: a comparative analysis with human H‐chain ferritin. J Mol Biol. 2005;347:543‐554. 15755449 10.1016/j.jmb.2005.01.007

[23] Torti FM , Torti SV . Regulation of ferritin genes and protein. Blood. 2002;99:3505‐3516. 11986201 10.1182/blood.v99.10.3505

[24] Arosio P , Levi S . Cytosolic and mitochondrial ferritins in the regulation of cellular iron homeostasis and oxidative damage. Biochim Biophys Acta. 2010;1800:783‐792. 20176086 10.1016/j.bbagen.2010.02.005

[25] Dodson M , Castro‐Portuguez R , Zhang DD . NRF2 plays a critical role in mitigating lipid peroxidation and ferroptosis. Redox Biol. 2019;23:101107. 30692038 10.1016/j.redox.2019.101107 PMC6859567

[26] Harada N , Kanayama M , Maruyama A , et al. Nrf2 regulates ferroportin 1‐mediated iron efflux and counteracts lipopolysaccharide‐induced ferroportin 1 mRNA suppression in macrophages. Arch Biochem Biophys. 2011;508:101‐109. 21303654 10.1016/j.abb.2011.02.001

[27] Bersuker K , Hendricks JM , Li Z , et al. The CoQ oxidoreductase FSP1 acts parallel to GPX4 to inhibit ferroptosis. Nature. 2019;575:688‐692. 31634900 10.1038/s41586-019-1705-2 PMC6883167

[28] Koppula P , Zhuang L , Gan B . Cystine transporter SLC7A11/xCT in cancer: ferroptosis, nutrient dependency, and cancer therapy. Protein Cell. 2021;12:599‐620. 33000412 10.1007/s13238-020-00789-5 PMC8310547

[29] Guo A , Zhang H , Li H , et al. Inhibition of connexin hemichannels alleviates neuroinflammation and hyperexcitability in temporal lobe epilepsy. Proc Natl Acad Sci. 2022;119:e2213162119. 36322757 10.1073/pnas.2213162119 PMC9659366

[30] Stockwell BR , Jiang X . The chemistry and biology of Ferroptosis. Cell Chem Biol. 2020;27:365‐375. 32294465 10.1016/j.chembiol.2020.03.013 PMC7204503

[31] Stockwell BR , Jiang X , Gu W . Emerging mechanisms and disease relevance of ferroptosis. Trends Cell Biol. 2020;30:478‐490. 32413317 10.1016/j.tcb.2020.02.009 PMC7230071

[32] Fuhrmann DC , Brüne B . A graphical journey through iron metabolism, microRNAs, and hypoxia in ferroptosis. Redox Biol. 2022;54:102365. 35717888 10.1016/j.redox.2022.102365 PMC9213245

[33] Hambright WS , Fonseca RS , Chen L , Na R , Ran Q . Ablation of ferroptosis regulator glutathione peroxidase 4 in forebrain neurons promotes cognitive impairment and neurodegeneration. Redox Biol. 2017;12:8‐17. 28212525 10.1016/j.redox.2017.01.021 PMC5312549

[34] Gao G , Chang Y . Mitochondrial ferritin in the regulation of brain iron homeostasis and neurodegenerative diseases. Front Pharmacol. 2014;5:19. 24596558 10.3389/fphar.2014.00019 PMC3925988

[35] Zhang N , Yu X , Xie J , Xu H . New insights into the role of ferritin in iron homeostasis and neurodegenerative diseases. Mol Neurobiol. 2021;58:2812‐2823. 33507490 10.1007/s12035-020-02277-7

[36] Kawabata H . Transferrin and transferrin receptors update. Free Radic Biol Med. 2019;133:46‐54. 29969719 10.1016/j.freeradbiomed.2018.06.037

[37] Vogt AS et al. On iron metabolism and its regulation. Int J Mol Sci. 2021;22:4591. 33925597 10.3390/ijms22094591 PMC8123811

[38] Yanatori I , Kishi F . DMT1 and iron transport. Free Radic Biol Med. 2019;133:55‐63. 30055235 10.1016/j.freeradbiomed.2018.07.020

[39] Liang P , Zhang X , Zhang Y , et al. Neurotoxic A1 astrocytes promote neuronal ferroptosis via CXCL10/CXCR3 axis in epilepsy. Free Radic Biol Med. 2023;195:329‐342. 36610561 10.1016/j.freeradbiomed.2023.01.002

[40] Cheng H , Wang P , Wang N , et al. Neuroprotection of NRF2 against Ferroptosis after traumatic brain injury in mice. Antioxidants. 2023;12:731. 36978979 10.3390/antiox12030731 PMC10044792

[41] Qin Z , Song J , Lin A , et al. GPR120 modulates epileptic seizure and neuroinflammation mediated by NLRP3 inflammasome. J Neuroinflammation. 2022;19:121. 35624482 10.1186/s12974-022-02482-2 PMC9137133

[42] Yang X , Cao Q , Guo Y , He J , Xu D , Lin A . GSDMD knockdown attenuates phagocytic activity of microglia and exacerbates seizure susceptibility in TLE mice. J Neuroinflammation. 2023;20:193. 37612735 10.1186/s12974-023-02876-w PMC10464294

[43] Chen S , Tsai CH , Lin CJ , et al. Transcranial focused ultrasound pulsation suppresses pentylenetetrazol induced epilepsy in vivo. Brain Stimul. 2020;13:35‐46. 31575487 10.1016/j.brs.2019.09.011

[44] Chen L , Xu Y , Cheng H , et al. Adult‐born neurons in critical period maintain hippocampal seizures via local aberrant excitatory circuits. Signal Transduct Target Ther. 2023;8:225. 37280192 10.1038/s41392-023-01433-4 PMC10244397

[45] Palmgren A . A rapid method for selective silver staining of nerve fibres and nerve endings in mounted paraffin sections. Acta Zool. 1948;29:377‐392.

