# Supplementary material for: Mitochondrial ferritin alleviates ferroptosis in a kainic acid‐induced mouse epilepsy model by regulating iron homeostasis: Involvement of nuclear factor erythroid 2‐related factor 2
Source: CNS Neurosci Ther. 2024 Mar 4;30(3):e14663. doi: 10.1111/cns.14663 (PMC10912846; doi:10.1111/cns.14663)
Supplement: Supplementary file 1 — Figures S1–S6 [file CNS-30-e14663-s001.docx]

**SUPPLEMENTTARY DATE**


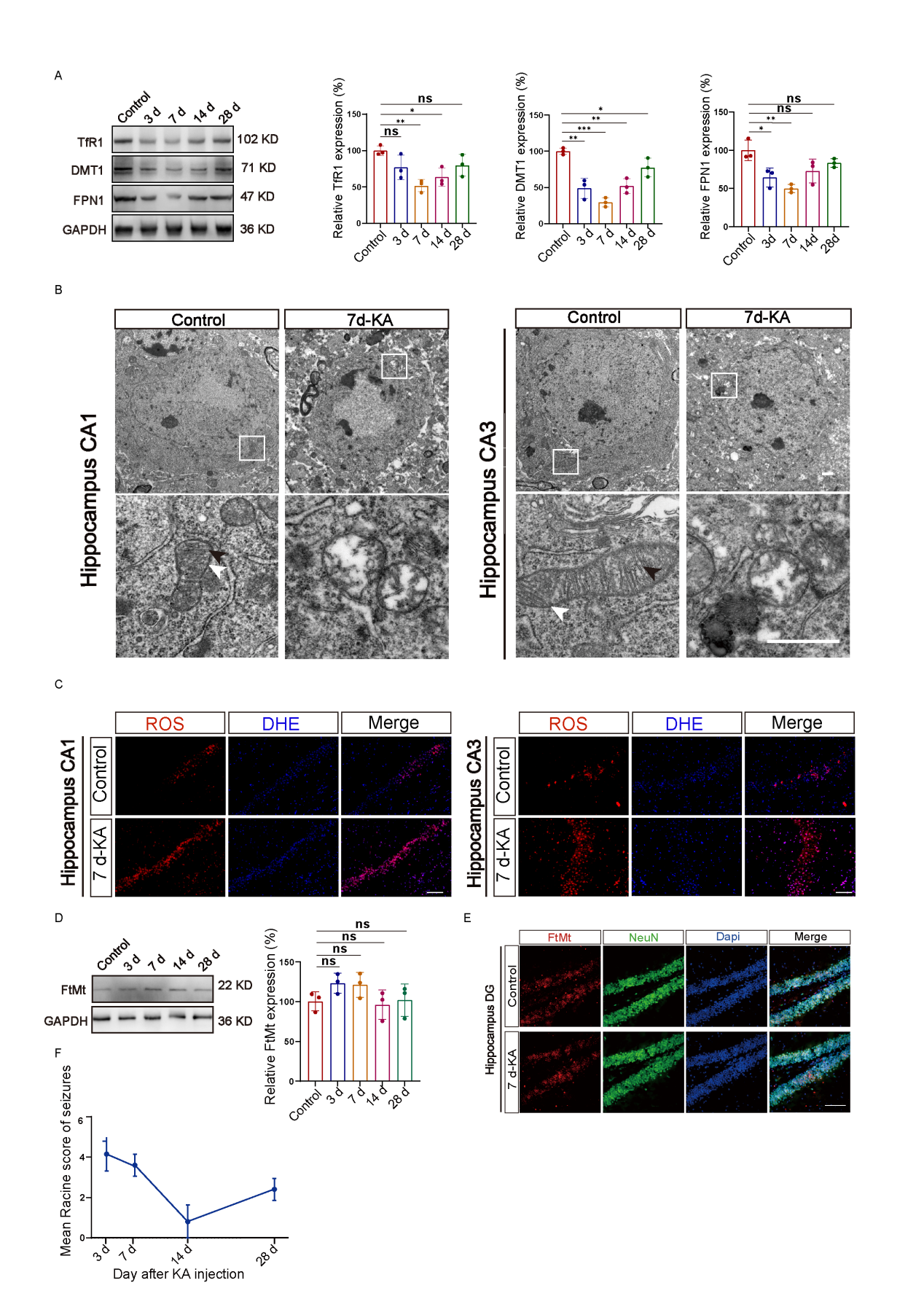


**Supplementary fig. 1** **A** Expression of iron ion transport-related proteins in the hippocampus of epileptic mice (n = 3 in each group). **B** Representative morphological changes to the mitochondria in different groups. Black arrows point to Mitochondrial crest. White arrows point to Mitochondrial bilayer membrane (scale bar = 100 μm). **C** ROS changes in the CA1 and CA3 regions of the hippocampus in epileptic mice (scale bar = 100 μm). **D** Expression of FtMt protein in the cortex of epileptic mice. **E** Immunofluorescence changes of FtMt protein in the intrahippocampal DG region of epileptic mice. **F** Racine grading of epileptic mice at different time points. All data are depicted with SEM. *P < 0.05 **P < 0.01, ***P < 0.001, ns not significant.

**
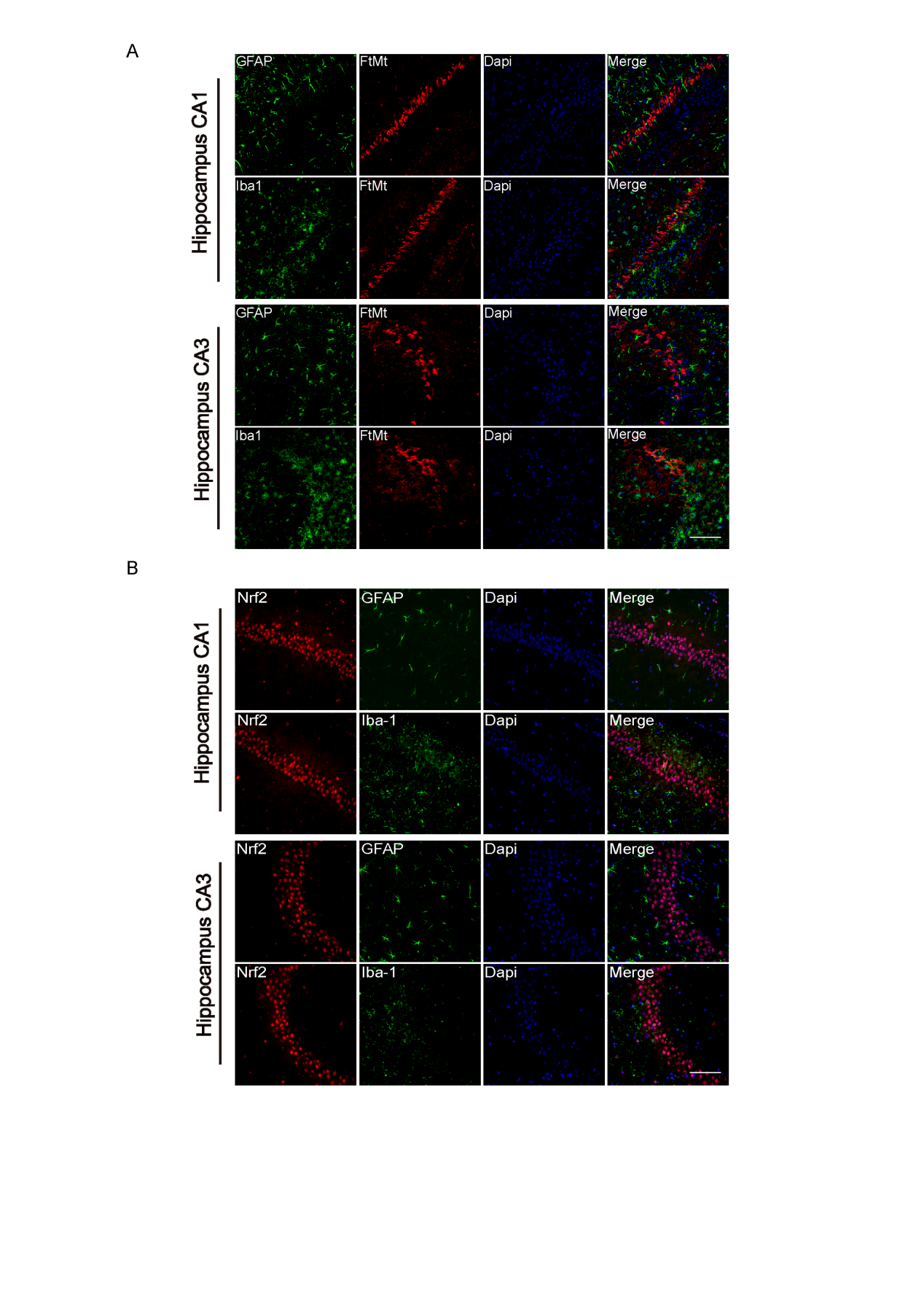
**

**Supplementary fig. 2** **A** FtMt did not co-localize with Iba1 or GFAP in hippocampal tissue of mice with epilepsy (scale bar = 100 μm). **B** Nrf2 did not co-localize with Iba1 or GFAP in the hippocampal tissue of mice with epilepsy (scale bar = 100 μm).

**
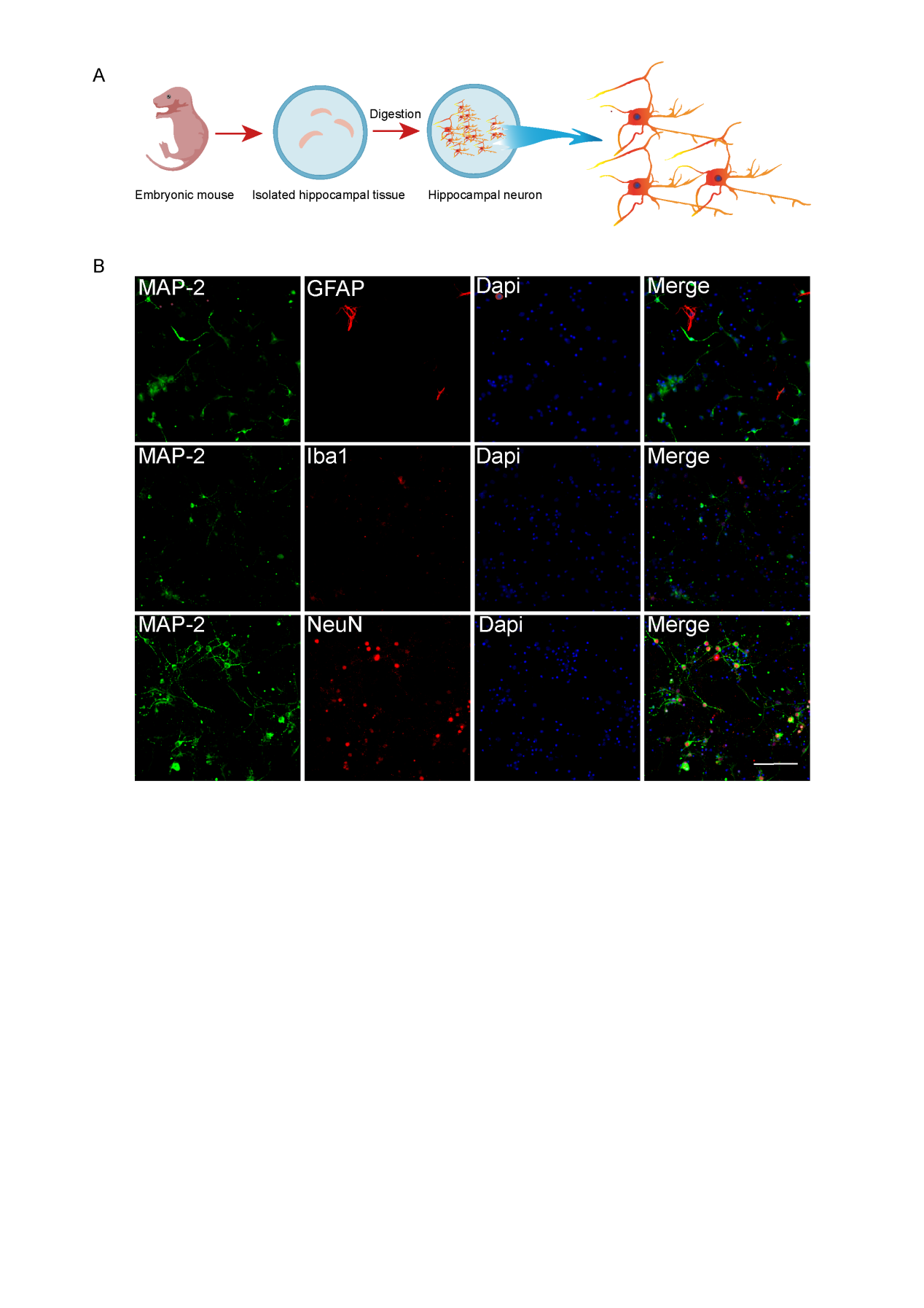
**

**Supplementary fig. 3** **A** Schematic diagram of the neuron extraction process. **B** Schematic diagram of neuron identification (scale bar = 100 μm).


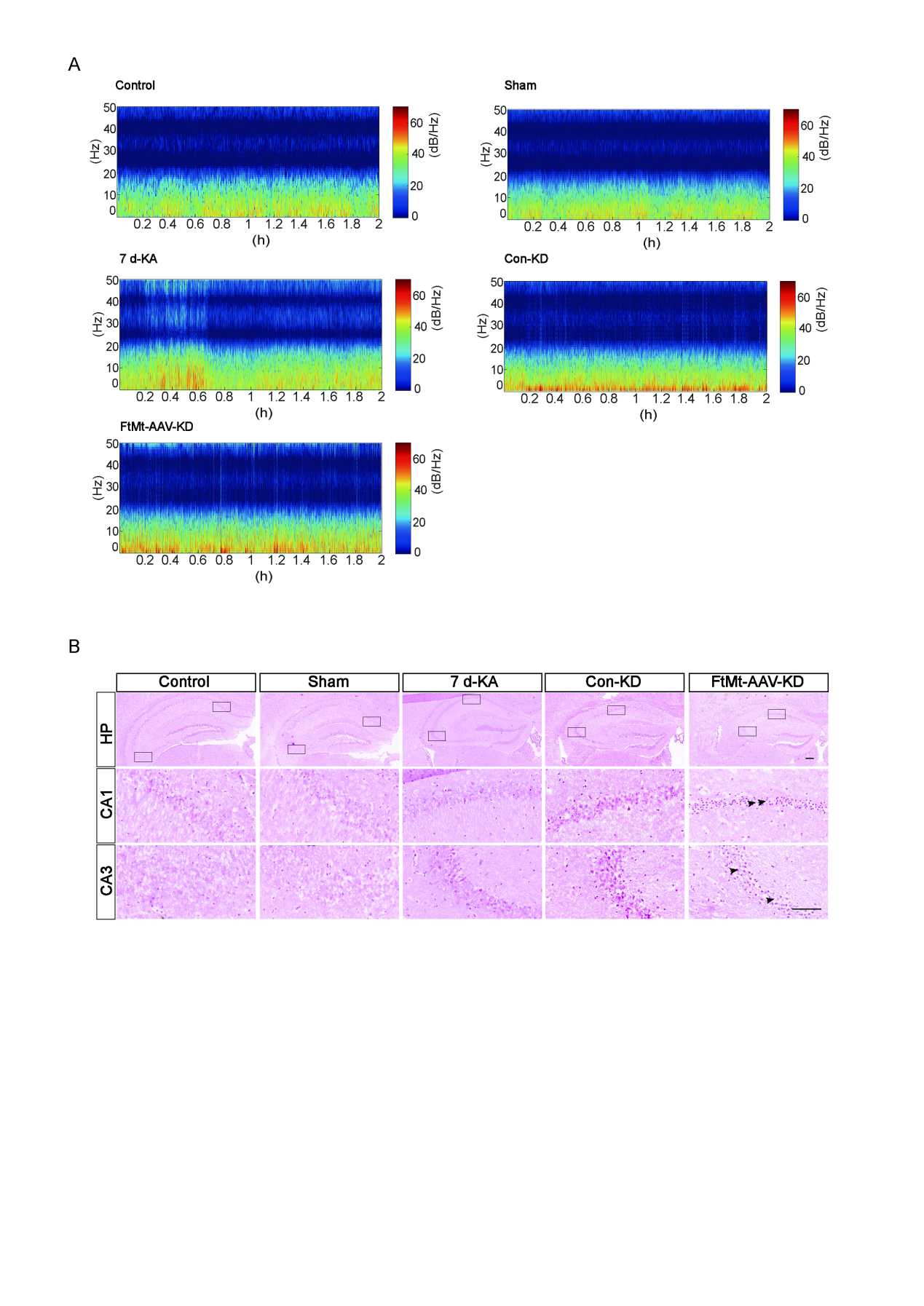


**Supplementary fig. 4** **A** Changes in FtMt in the hippocampus of epileptic mice affect their brain power spectrum. **B** Lillie were used to measure Fe^2+^ in the hippocampal CA1 and CA3 regions (scale bar = 100 μm). Arrows point to Fe^2+^.

**Supplementary fig. 5**

Real-time PCR primers used for quantification of mRNA expression in this study

Primer name Sequence ((5′ → 3′)

| *Ptgs2* | Forward | TTCAACACACTCTATCACTGGC |
| --- | --- | --- |
|  | Reverse | AGAAGCGTTTGCGGTACTCAT |
| GAPDH | Forward | AGGTCGGTGTGAACGGATTTG |
|  | Reverse | TGTAGACCATGTAGTTGAGGTCA |

**Supplementary fig. 6**

rAAV-U6-shRNA (*FtMt*)-CMV-EGFP-pA primers used in this study

Primer name Sequence ((5′ → 3′)

| Forward | CAUCAAGAAGCCAGAUAAATT |
| --- | --- |
| Reverse | UUUAUCUGGCUUCUUGAUGTT |

*FtMt* siRNA primers used in this study

Primer name Sequence ((5′ → 3′)

| Forward | CAUCAAGAAGCCAGAUAAATT |
| --- | --- |
| Reverse | UUUAUCUGGCUUCUUGAUGTT |

pAAV-CMV-Nfe212-3xFLAG-WPRE primers used in this study

Primer name Sequence ((5′ → 3′)

| Forward | CGCAAATGGGCGGTAGGCGTG |
| --- | --- |
| Reverse | CATAGCGTAAAAGGAGCAACA |
